# Endothelin receptors promote schistosomiasis-induced hepatic fibrosis via splenic B cells

**DOI:** 10.1371/journal.ppat.1008947

**Published:** 2020-10-19

**Authors:** Hongyan Kong, Jinan He, Shusen Guo, Qiqin Song, Dandan Xiang, Ran Tao, Haijing Yu, Guang Chen, Zhiyong Huang, Qin Ning, Jiaquan Huang

**Affiliations:** 1 Department and Institute of Infectious Disease, Tongji Hospital, Tongji Medical College, Huazhong University of Science and Technology, Wuhan, China; 2 Department of Pediatrics, Tongji Hospital, Tongji Medical College, Huazhong University of Science and Technology, Wuhan, China; 3 Hepatic Surgery Center, Tongji Hospital, Tongji Medical College, Huazhong University of Science and Technology, Wuhan, China; University of Manchester, UNITED KINGDOM

## Abstract

Endothelin receptors (ETRs) are activated by vasoactive peptide endothelins and involved in the pathogenesis of hepatic fibrosis. However, less is known about the role of ETRs in *Schistosoma (S*.*) japonicum*-induced hepatic fibrosis. Here, we show that the expression of ETRs is markedly enhanced in the liver and spleen tissues of patients with schistosome-induced fibrosis, as well as in murine models. Additional analyses have indicated that the expression levels of ETRs in schistosomiasis patients are highly correlated with the portal vein and spleen thickness diameter, both of which represent the severity of fibrosis. Splenomegaly is a characteristic symptom of schistosome infection, and splenic abnormality may promote the progression of hepatic fibrosis. We further demonstrate that elevated levels of ETRs are predominantly expressed on splenic B cells in spleen tissues during infection. Importantly, using a well-studied model of human schistosomiasis, we demonstrate that endothelin receptor antagonists can partially reverse schistosome-induced hepatic fibrosis by suppressing the activation of splenic B cells characterized by interleukin-10 (IL-10) secretion and regulatory T (Treg) cell-inducing capacity. Our study provides insights into the mechanisms by which ETRs regulate schistosomiasis hepatic fibrosis and highlights the potential of endothelin receptor antagonist as a therapeutic intervention for fibrotic diseases.

## Author summary

Schistosomiasis is a serious but neglected tropical infectious disease. which can lead to hepatic fibrosis and death. To date, there are still no approved antifibrotic therapies. Hepatic fibrosis results in portal hypertension and variceal bleeding, and it is the primary cause of mortality from schistosomiasis. Splenomegaly and hypersplenism can manifest following the development of portal hypertension. Accumulating evidence suggests that the spleen plays a critical role in the development of hepatic fibrosis. In this study, using *Schistosoma (S*.*) japonicum* in both humans and mice, we show that progressive hepatic schistosomiasis caused elevation of endothelin receptors (ETRs) both in liver and spleen tissues, and the endothelin receptor-producing cells are mainly located in splenic B cells. More importantly, we demonstrate that endothelin receptor antagonists can partially reverse schistosome-induced hepatic fibrosis by suppressing the activation of splenic B cells during infection. Thus, our study highlights the potential of endothelin receptor antagonist as a therapeutic intervention for schistosomiasis and other fibrotic diseases.

## Introduction

Schistosomiasis is a serious parasitic disease throughout the world’s tropical regions, affecting more than 200 million people worldwide [1]. Schistosome worms lay their eggs in the mesenteric and portal veins of their human host, and the eggs are trapped in liver sinusoids [2]. Here, larval miracidia within the mature eggs secrete toxins that elicit host immune responses including granulomatous inflammation and fibrotic reactions [3]. Intestinal and hepatic schistosomiasis are the most common forms of chronic disease. Intestinal schistosomiasis is an acute or chronic, specific enteropathy caused by the deposition of schistosome ovum on the colon and rectal walls [4]. Gastrointestinal symptoms may include abdominal pain, altered bowel habits, and bloody stools [5]. The egg-induced hepatic fibrosis, which can lead to portal hypertension and variceal bleeding, is the primary cause of morbidity and mortality associated with this chronic disease [6]. Several studies by our and other groups have shown that the regulators of schistosomiasis hepatic fibrosis are far more complicated than expected[7, 8]. Splenomegaly is a consequence and an important clinical indicator of portal hypertension [9]. Previous studies have suggested that splenic abnormalities may promote the progression of liver fibrosis to cirrhosis and exacerbate disease prognosis through multiple possible pathways [10, 11]. However, the role of the spleen in severe schistosomiasis has been little explored and probably involves more than just a contribution to portal blood hypertension [12]. Although chemotherapy can effectively target and kill schistosomes, the progression of hepatic fibrosis persists [13]. Since anti-fibrotic therapies for schistosomiasis have been neglected, new prospective drugs are urgently required that can reverse the fibrosis.

Endothelin receptors (ETRs) are class A G-protein-coupled receptors (GPCRs) activated by the vasoactive peptide hormones endothelins [14]. Two subtypes of ETRs (ETAR and ETBR) [15, 16] are involved in various functions, such as regulation of blood pressure, sodium excretion, cell proliferation, and neural crest development [17, 18]. Endothelin-1 (ET-1) is a peptide that is produced primarily by vascular endothelial cells and is characterized as a powerful smooth muscle vasoconstrictor and mitogen [19]. In addition, autocrine and paracrine signaling of ET-1 exerts its effects by binding to ETRs, thus triggering the downstream signaling in cells [20]. Both receptors are found in vascular smooth muscle where they mediate vasoconstriction, whereas in the endothelium, ETBR mediates vasodilatation in part through nitric oxide release [21]. Studies have found that ET-1 and its receptors are related to liver fibrosis or cirrhosis. Plasma ET-1 levels are increased in cirrhosis and correlate with the severity of liver disease and portal pressure [22]. Several studies by our and other groups have shown that elevated concentrations of ET-1 act on upregulated ETRs on hepatic stellate cells (HSCs) to cause increased contractility and intrahepatic sinusoidal resistance, resulting in portal hypertension [23, 24]. The spleen plays a critical role in development of liver fibrosis and cirrhosis [25]. A previous study has shown that the high concentration of ET-1 in cirrhosis with portal hypertension could be due to increased splenic production of this endothelial factor [26]. The splenic cells involved in the increased endothelin production are endothelial cells of the vein sinuses and B-cells in the germinal centers and in the marginal zone of lymphoid sheaths and follicles [27]. These findings prompted us to ask whether the splenocytes express ETRs and are involved in the progression of hepatic fibrosis or cirrhosis. In addition, the role of ETRs in schistosomiasis has been little explored, and no study thus far has localized ETAR and ETBR in *Schistosoma (S*.*) japonicum*-infected spleen tissues.

As the largest lymphoid organ in the body, the spleen contains highly elaborate tissue structures and is anatomically linked to the liver via the portal vein system [28]. B cells, representing a major immune cell population, are well known for their ability to differentiate into antibody-secreting plasma cells in response to foreign antigens or pathogens [29]. However, recent studies have suggested that B cells appear to regulate both protective and pathologic immune responses by antibody-independent mechanisms [30]. Helminth infections, particularly infection with schistosomes, are well-known to induce regulatory B cells [31–33], a relatively new member of the network of regulatory immune cells. B cells suppress pro-inflammatory immune responses via several mechanisms, of which the best described are the expression of the regulatory cytokine interleukin-10 (IL-10) and induction of regulatory T (Treg) cells [34].

In the current study, we used both human schistosomiasis and a mouse model to investigate the role of ETAR and ETBR. We hypothesized that ETRs play an important role in the pathogenesis of schistosomiasis. We attempted to apply endothelin receptor antagonists, including BQ-123, a selective ETA antagonist, and BQ-788, a selective ETB antagonist, to demonstrate our hypothesis and further elucidate the molecular pathogenesis pathways through which ETRs may cause fibrotic changes in the spleens of humans and mice upon exposure to *S*. *japonicum* infection.

## Results

### Enhanced expression of ETRs in liver tissues of patients with schistosome-induced hepatic fibrosis

**Fig 1:**
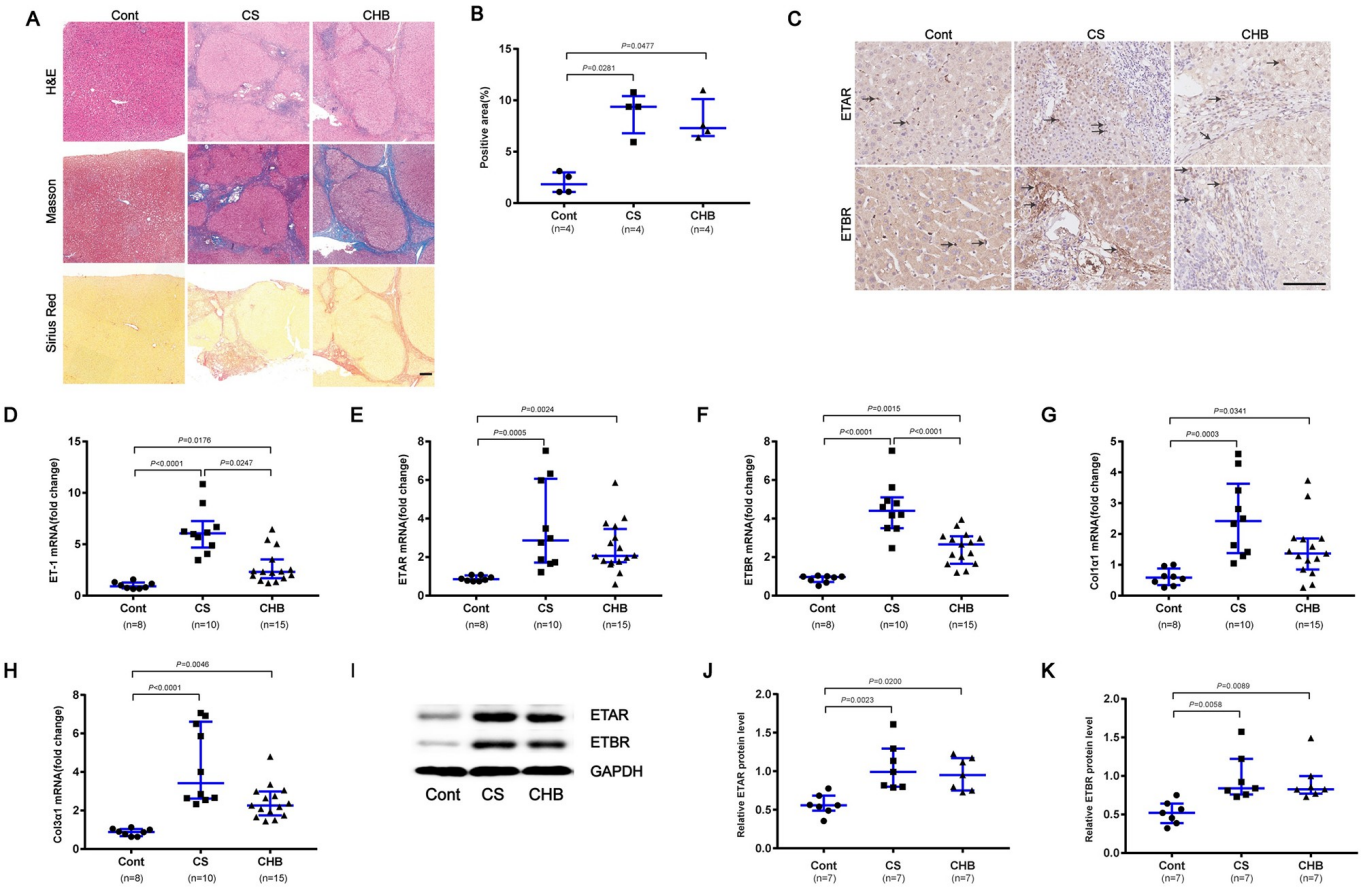
Enhanced expression of ETRs in liver tissues of patients with schistosome-induced hepatic fibrosis. (A) Paraffin-embedded sections of liver tissues from patients were stained with H&E, Masson’s trichrome and Sirius Red. Scale bar, 200 μm. (B) The positive staining areas for Sirius Red staining were measured using IPP software (n = 4). (C) Representative immunohistochemical staining of ETAR and ETBR. Black arrows indicate the ETRs positive cells. Scale bar, 100 μm. (D-H) Total RNA was extracted for qPCR analysis of ET-1, ETAR, ETBR, Col1α1 and Col3α1 expression levels. Cont (n = 8), CS (n = 10), CHB (n = 15). (I-K) ETAR and ETBR proteins were determined by western blotting. Image density was quantified using Image J analysis and normalized to GAPDH (n = 7). Data are represented as the median and interquartile range. All data are representative of at least two independent experiments. Significance was calculated using Kruskal–Wallis with Dunn’s posttest (B, D-H, J-K). Abbreviation: Cont, control; CS, chronic schistosomiasis; CHB, chronic hepatitis B.

To detect the expression of ETRs and fibrotic molecules in liver specimens, and analyse their correlations with the progression of schistosome-induced hepatic fibrosis, liver biopsy was performed in chronic schistosomiasis (CS) patients, chronic hepatitis B (CHB) patients and control (Cont) patients. Compared with Cont human liver tissues, fibrotic liver tissues had larger fibrotic areas and more collagen deposition, as represented by Sirius Red staining, and *S*. *japonicum* eggs were observed in liver specimen sections of patients with CS (Fig 1A and 1B). To evaluate the functional effects of ETRs, we analyzed ETAR and ETBR in liver tissues by immunohistochemistry. Both the CHB and CS groups showed stronger ETAR and ETBR staining than control liver tissues (Fig 1C). Representative photomicrographs revealed that pathological lesions and collagen deposition in liver biopsy samples of patients with CS significantly increased with the aggravation of liver fibrosis compared with S0 (S1A and S1B Fig). ETAR and ETBR-positive cells were localized mainly in areas of inflammation and fibrosis (S1C Fig). Our data revealed that the mRNA expression level of ET-1, ETAR, ETBR, Col1α1 and Col3α1 was significantly higher in the fibrotic groups than the control group (Fig 1D–1H). In addition, the schistosome-induced liver fibrosis group appeared to have slightly stronger ET-1 and ETBR expression than the HBV-induced group (Fig 1D and 1F). Furthermore, immunoblots were analyzed and confirmed the transcriptional levels of ETAR and ETBR (Fig 1I–1K).

### Enhanced expression of ETRs in spleen tissues of patients with schistosome-induced hepatic fibrosis

**Fig 2:**
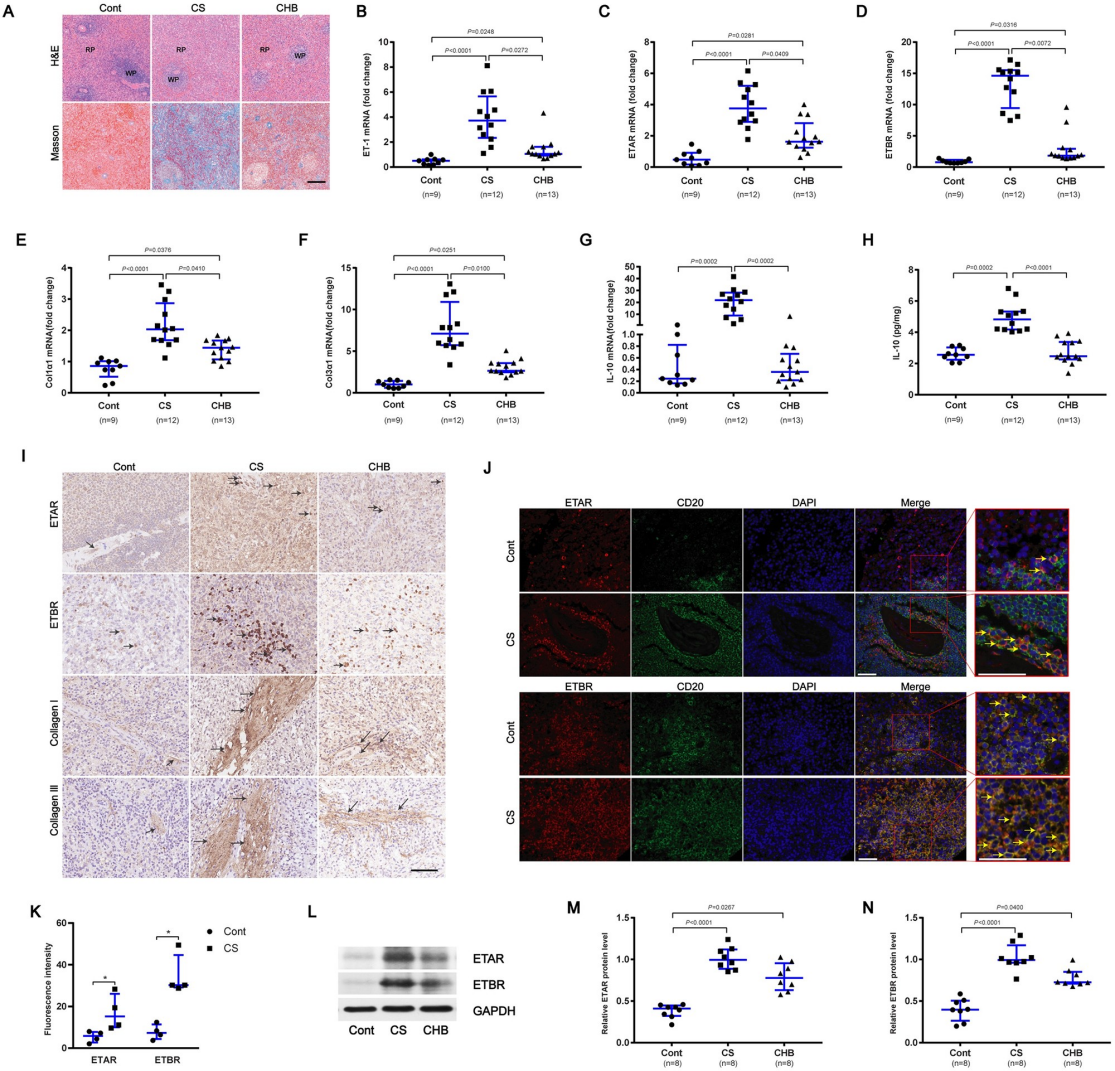
Enhanced expression of ETRs in spleen tissues of patients with schistosome-induced hepatic fibrosis. (A) The human spleen tissues were stained with H&E and Masson’s trichrome. Scale bar, 200 μm. (B-G) qPCR analysis of the expression levels of ET-1, ETAR, ETBR, Col1α1, Col3α1 and IL-10 in spleen samples. Cont (n = 9), CS (n = 12), CHB (n = 13). (H) The IL-10 concentration in spleen tissue homogenates was determined by ELISA. Cont (n = 9), CS (n = 12), CHB (n = 13). (I) Representative immunohistochemical staining of ETAR, ETBR, collagen I and collagen III. Black arrows indicate the ETRs, collagen I and collagen III positive cells. Scale bar, 100 μm. (J) Representative immunofluorescence staining of ETAR, ETBR and CD20^+^ B cells in human spleen tissues. *Insets* show a higher magnification of the outlined area. Yellow arrows denote positive cells. Scale bar, 50 μm. (K) The fluorescence intensity was measured using IPP software (n = 4). (L-N) ETAR and ETBR proteins were determined by western blotting, quantified using Image J analysis, and normalized to GAPDH (n = 7). Data are represented as the median and interquartile range from two independent experiments. Multiple comparisons were performed by Kruskal–Wallis with Dunn’s posttest (B-H, M-N) or the Mann-Whitney U-test (K). *P < 0.05. Abbreviation: Cont, control; CS, chronic schistosomiasis; CHB, chronic hepatitis B; RP: red pulp; WP: white pulp.

To further investigate the role of ETRs in liver fibrosis, we collected human spleen tissues of patients with Cont, CS and CHB. In CS and CHB spleen tissues, splenic red pulp was dilated by congestion, and the size of the splenic white pulp was reduced and collagen deposition was increased compared with Cont tissues (Fig 2A). We found the mRNA levels of ET-1, ETAR, ETBR, Col1α1 and Col3α1 were upregulated in both patients with CS and CHB, with a greater increase in CS patients (Fig 2B–2F). In contrast, the level of IL-10 was only increased in patients with CS but not CHB spleen tissues compared with control tissues (Fig 2G). The IL-10 concentration in spleen tissue homogenates, as detected by ELISA, was found to be upregulated in CS tissues compared with CHB and Cont (approximately 2.4-fold) (Fig 2H). The mRNA levels of ETAR and ETBR were confirmed by immunoblotting (Fig 2L–2N). The immunohistochemistry study revealed enhanced ETAR, ETBR, collagen I and collagen III in spleen tissues from patients with CS and CHB (Fig 2I). Double staining using immunofluorescence displayed a co-localization of ETRs and CD20 staining, indicating that human splenic B cells express ETAR and ETBR in vivo (Fig 2J). Compared with control human spleen, the elevated levels of ETAR (2.6-fold) and ETBR (4.2-fold) were predominantly expressed on splenic B cells in spleen tissues of patients with schistosome-induced hepatic fibrosis (Fig 2K), while they were scarce on endothelial cells of the splenic sinus (S2 Fig).

### Comparison of the clinical indicators and ETRs are correlated with fibrogenesis

**Fig 3:**
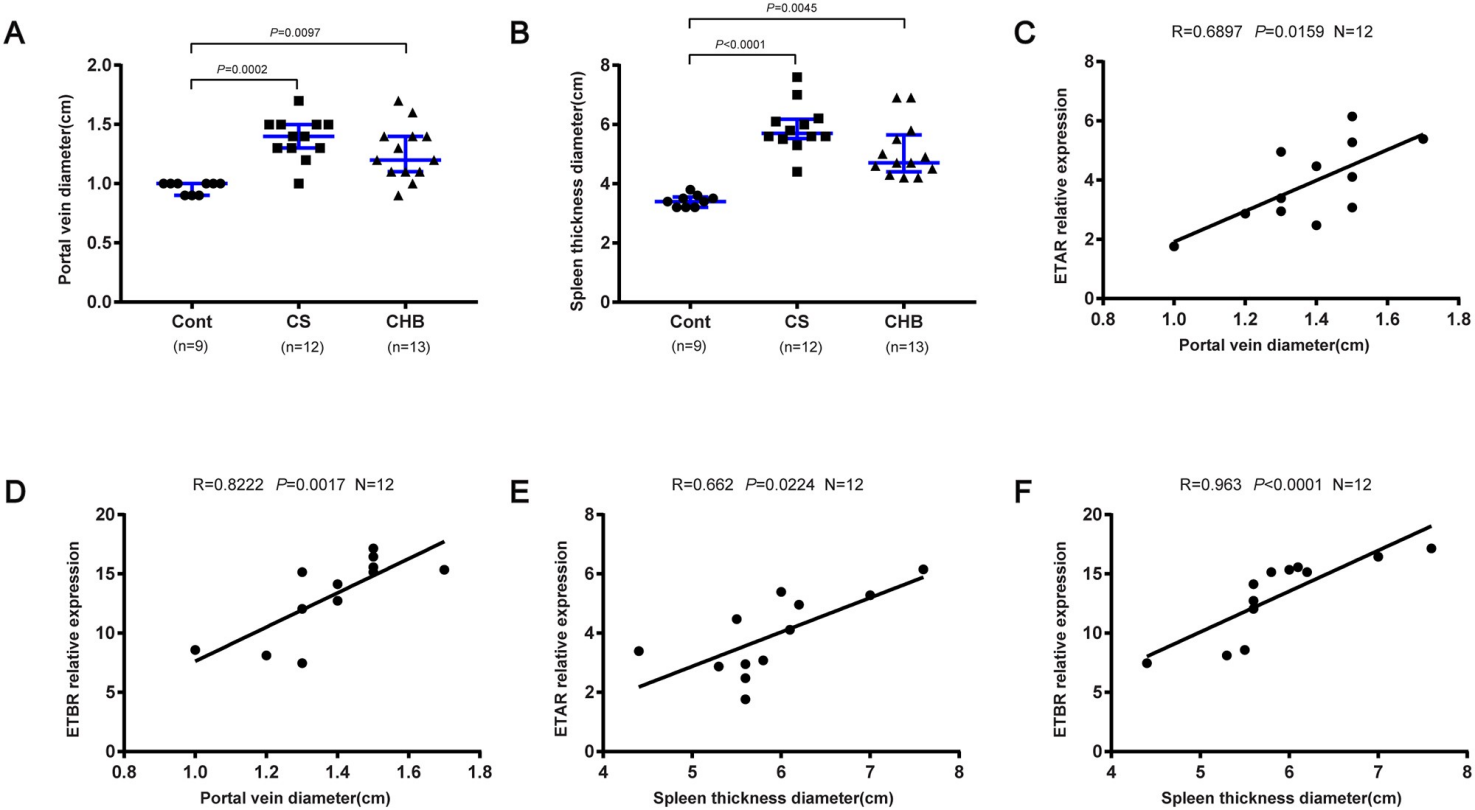
ETAR and ETBR were correlated with fibrogenesis. (A-B) Portal vein and spleen thickness diameter in patients who underwent splenectomy. Cont (n = 9), CS (n = 12), CHB (n = 13). (C-D) Correlations between mRNA levels of ETAR and ETBR with portal vein diameter in CS patients (n = 12); (E-F) Correlations between mRNA levels of ETAR and ETBR with spleen thickness diameter in CS patients (n = 12). Data are represented as the median and interquartile range (A-B). Significance was calculated using Kruskal–Wallis with Dunn’s posttest (A-B) or Spearman rank test (C-F). Abbreviation: Cont, control; CS, chronic schistosomiasis; CHB, chronic hepatitis B.

Next, we investigated some clinical parameters in 34 patients of 3 groups who underwent splenectomy. Abdominal ultrasonography indicated that the portal vein diameter and spleen thickness diameter were remarkably higher in CS and CHB patients than Cont (Fig 3A and 3B), while the expression levels of ALT, AST, TBIL, ALP and GGT showed no significant differences among the 3 groups (S3A–S3E Fig). In contrast, the level of PLT was decreased in CS and CHB patients than Cont (S3F Fig). To better define the roles of ETAR and ETBR in fibrotic progression, we compared the mRNA levels of ETAR and ETBR in CS patients using imaging tests (e.g., portal vein diameter and spleen thickness diameter). The mRNA levels of ETAR and ETBR were positively correlated with the portal vein diameter and spleen thickness diameter in patients with CS (Fig 3C–3F).

### Enhanced expression of ETRs in murine schistosome-induced hepatic fibrosis

**Fig 4:**
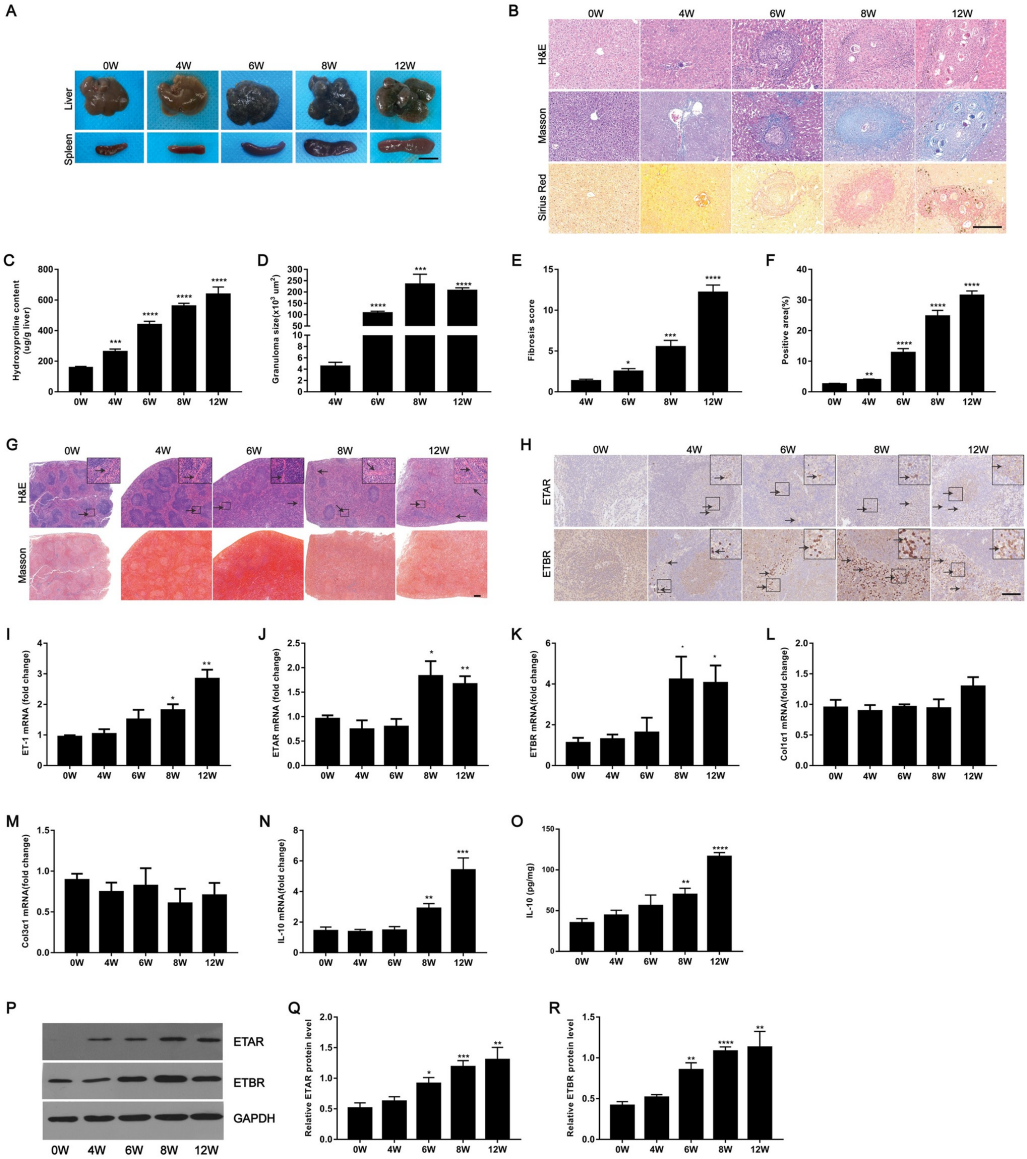
Analysis of ETRs expression in murine schistosomiasis. (A) Micrographs of livers and spleens from different weeks after infection. Scale bar, 1 cm. (B) H&E, Masson’s trichrome and Sirius Red staining of liver sections. Scale bar, 200 μm. (C) Collagen content in livers determined as hydroxyproline content (n = 6). (D) Granuloma size measured from H&E-stained liver sections(n = 6). (E) Fibrosis scores measured from Masson’s trichrome staining of liver sections (n = 6). (F) Positive staining areas for Sirius Red were measured using IPP software (n = 6). (G) H&E and Masson’s trichrome staining of spleen sections. *Insets* show a higher magnification of the outlined area. Black arrows indicate the Trabeculae. Scale bar, 200 μm. (H) Representative immunohistochemical staining for ETAR and ETBR in the infected spleens. *Insets* show a higher magnification of the outlined area. Black arrows indicate the ETRs positive cells. Scale bar, 100 μm. (I-N) The expression of ET-1, ETAR, ETBR, Col1α1, Col3α1 and IL-10 in spleens during infection was detected by qPCR (n = 3–6). (O) The IL-10 concentration in spleen tissue homogenates was determined by ELISA (n = 6). (P-R) ETAR and ETBR proteins were determined by western blotting, quantified using Image J analysis, and normalized to GAPDH (n = 5). Data are represented as mean ± SEM. All data are representative of at least three independent experiments. Significance was determined by the two-tailed Student’s t test (C-F, I-O, Q-R). *P < 0.05, **P< 0.01, ***P< 0.001, ****P< 0.0001, compared with 0W samples (C, F, I-O, Q-R) and compared with 4W samples (D-E).

To further investigate the role of endothelin receptor in schistosomiasis, we applied the well-established model of *S*. *japonicum*-induced for hepatic fibrogenesis in mice and evaluated their expression during the progression of hepatic schistosomiasis. Liver and spleen samples were harvested at 4, 6, 8 and 12 weeks post-infection from mice infected with *S*. *japonicum*. The results showed that we had successfully established a mouse model of *S*. *japonicum* infection in which hepatic injury and spleen enlargement could be observed (Fig 4A). These results were confirmed by the elevation level of alanine aminotransferase (ALT) and aspartate aminotransferase (AST) in mouse serum and further confirmed by the liver and spleen indexes (S4E–S4H Fig). Portal hypertension is a characteristic manifestation of advanced *S*. *japonicum* infection. The results showed that liver portal vein pressure and diameter were significantly increased after schistosome infection (S4B–S4D Fig). Importantly, hydroxyproline quantification, Masson’s trichrome and Sirius Red staining revealed that *S*. *japonicum* infection induced progressive hepatic injury and collagen deposition (Fig 4B, 4C, 4E and 4F), and the size of the hepatic granulomas was increased, as shown by H&E staining (Fig 4B and 4D). These results were confirmed by enhanced expression of ET-1, ETAR, ETBR, Col1α1 and Col3α1 over the course of *S*. *japonicum* infection in mice (S4A and S4I–S4P Fig). Given that splenomegaly is considered a prominent indication of liver fibrosis due to *S*. *japonicum*, we further examined the levels of splenic fibrosis in our model. Microscopic findings of spleen sections of *S*. *japonicum* showed congestion of red blood cells and disruption of lymphoid follicles. Increases in trabeculae and sinus hyperplasia were also noted (Fig 4G). However, collagen deposition was not significantly altered by Masson’s trichrome staining in these mice (Fig 4G). The protein levels of ETAR and ETBR determined by immunohistochemistry and western blotting were increased in spleen tissues during infection compared with uninfected tissues (Fig 4H and 4P–4R). The mRNA expression levels of ET-1, ETAR, ETBR, and IL-10 in infected spleen tissues were elevated (Fig 4I–4K and 4N), while the levels of Col1α1 and Col3α1 were unchanged during the observation period (Fig 4L and 4M). In addition, at 8 weeks and 12 weeks post-infection, significantly increased IL-10 secretion was measured in spleen tissue homogenates (2.0 to 3.3-fold) (Fig 4O).

### Endothelin receptor antagonists partially reverse schistosome-induced hepatic fibrosis

**Fig 5:**
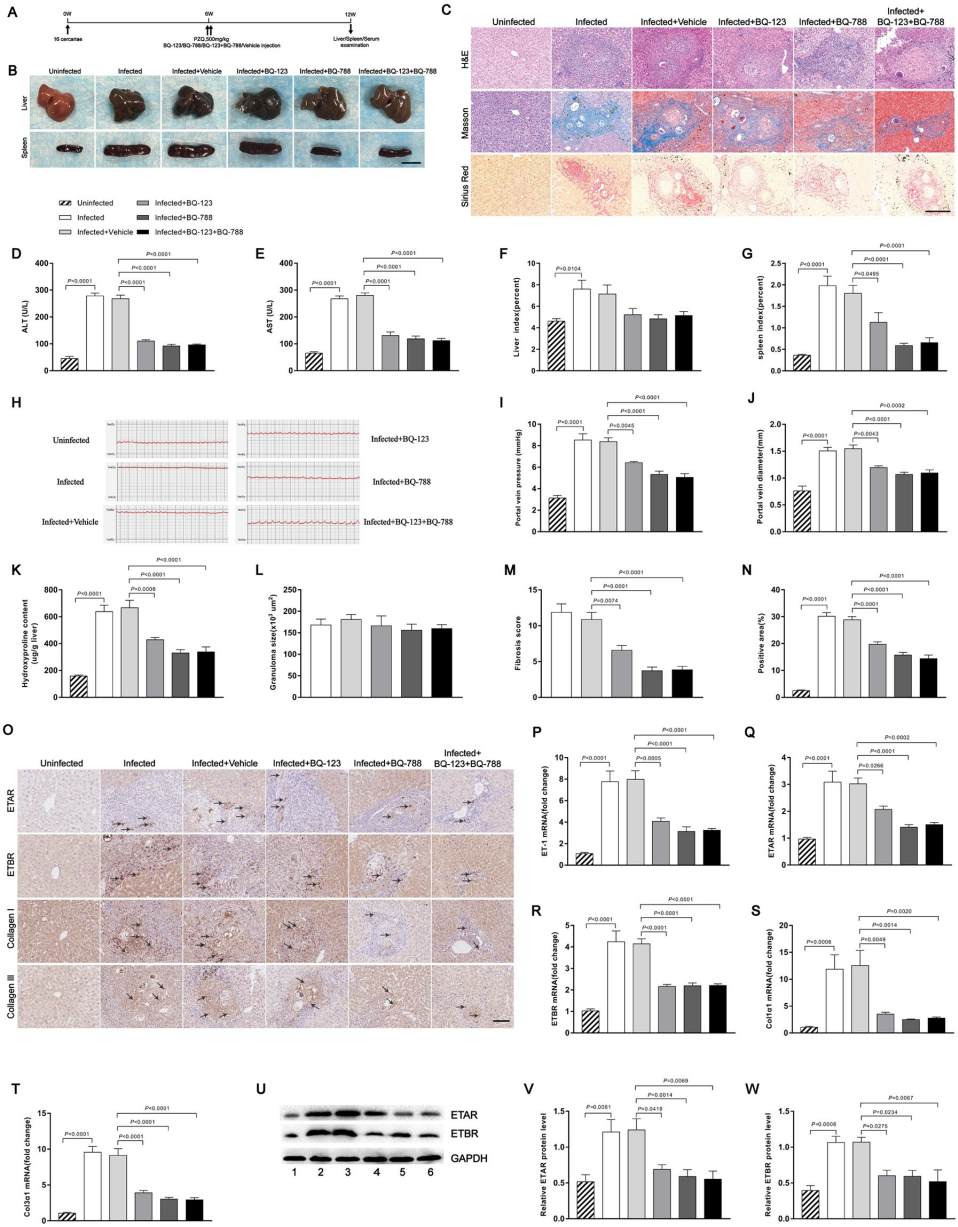
Endothelin receptor antagonists mediated reduction of hepatocellular damage. Mice were infected percutaneously with 16 *S*. *japonicum* cercariae or remained uninfected. At 6 weeks post-infection, all infected mice were treated with praziquantel to kill the parasites and then received either endothelin receptor antagonists or vehicle and were necropsied at 12 weeks post-infection. (A) Time schedule for parasite infection and administration of anti-parasite drug or endothelin receptor antagonists and sample withdrawal. (B) Macrograph of livers and spleens from uninfected mice, infected mice and infected mice treated with endothelin receptor antagonists. Scale bar, 1 cm. (C) H&E, Masson’s trichrome and Sirius Red staining of liver sections. Scale bar, 200 μm. (D-E) Serum ALT and AST levels were measured (n = 5–7). (F-G) Liver and spleen indexes were determined (n = 7). (H) Measurement of hepatic portal venous pressure in vivo by RM6240BD. (I) Statistical analysis of hepatic portal vein pressure (n = 7). (J) Analysis of the portal vein diameter in vivo (n = 7). (K) Collagen content in livers determined as hydroxyproline content (n = 6). (L) Granuloma size measured based on H&E staining of liver sections (n = 7). (M) Fibrosis scores measured based on Masson’s trichrome staining of liver sections (n = 7). (N) The positive staining areas for Sirius Red were measured using IPP software (n = 7). (O) Representative immunohistochemical staining for ETAR, ETBR, collagen I and collagen III in infected livers. Black arrows indicate the ETRs, collagen I and collagen III positive cells. Scale bar, 100 μm. (P-T) qPCR analysis of the expression levels of ET-1, ETAR, ETBR, Col1α1 and Col3α1 in liver samples (n = 6). (U-W) ETAR and ETBR proteins were determined by western blotting. 1, uninfected; 2, infected; 3, infected + vehicle; 4, infected + BQ-123; 5, infected + BQ-788; 6, infected + BQ-123 + BQ-788. Image density was quantified using Image J analysis and normalized to GAPDH (n = 5). Data are represented as mean ± SEM of three independent experiments. Multiple comparisons were performed by one-way ANOVA with Tukey’s correction for comparison between two groups (D-G, I-N, P-T, V-W). Abbreviation: ALT: alanine aminotransferase; AST: aspartate aminotransferase.

To investigate whether endothelin receptor antagonists can reverse the egg-inducing hepatic fibrosis, mice were infected with a moderate dose of parasites. At 6 weeks post-infection, when hepatic fibrosis was clearly manifested, all infected mice were treated with praziquantel to kill the parasite and then injected with either endothelin receptor antagonists or vehicle and necropsied at 12 weeks post-infection (Fig 5A). Consistent with previous study, praziquantel significantly reduced the worm burden (0.3-fold) and egg count (0.4-fold) (S5A–S5D Fig). Morphological changes in liver and spleen samples showed a moderate granulomatous response and splenomegaly in the endothelin receptor antagonist-treated mice (Fig 5B). Although the liver index of the mice in the treated groups appeared to have a slight reduction compared with the vehicle group, the difference was not statistically significant (Fig 5F). The spleen index was significantly reduced with anti-fibrosis treatment (Fig 5G). The results revealed that mice treated with endothelin receptor antagonists showed significant reductions in circulating levels of ALT and AST, suggesting the treatment alleviated hepatocellular damage (Fig 5D and 5E). Mice receiving endothelin receptor antagonists displayed a significant reduction in liver portal vein pressure and diameter (Fig 5H–5J), as well as extracellular matrix (ECM) deposition as shown by hydroxyproline quantification (Fig 5K), Masson’s trichrome and Sirius Red staining (Fig 5C, 5M and 5N), whereas the size of the hepatic granulomas in all groups was similar as shown by H&E staining (Fig 5L) and livers showed no significant change in egg burden (S6D Fig). Morphometric analysis of the liver tissues showed a healthier hepatic parenchyma with fewer fibrotic areas in mice treated with endothelin receptor antagonists (S6A and S6B Fig). Reduction of fibrosis was further confirmed by qPCR-based quantification of fibrosis-associated gene expression in the livers of infected mice. The amounts of ET-1, ETAR, ETBR, Col1α1 and Col3α1 mRNA were dramatically reduced in livers of mice treated with both BQ-123 and BQ-788 (Fig 5P–5T). Immunohistochemical staining and western blot analysis confirmed the reduced expression levels of ETRs and fibrosis markers in the treated mice (Fig 5O and 5U–5W). Consistent with previous study [35], the result suggested that the enhancements of ETRs in the infected mice were effectively inhibited by the endothelin receptor antagonists. In addition, we found that the serum level of SEA-specific IgG was decreased in the presence of BQ-788 and BQ-123 + BQ-788 (0.5-fold). Although the BQ-123-treated group appeared to display a slight reduction of SEA-IgG compared with the vehicle group, the difference was not statistically significant (0.7-fold) (S6C Fig). This suggested that ETRs were important for eliciting the humoral immune response after *S*. *japonicum* infection. Furthermore, we also found lack of significant differences in the levels of IFN-γ, TNF, IL-2, IL-4, and IL-5 in serum among the groups, while the level of IL-13 was decreased after treated with endothelin receptor antagonists (0.4 to 0.7-fold) (S7A–S7F Fig). These results suggested that endothelin receptor antagonists could partially alter Th1/Th2 responses to schistosome parasite infection in mice.

**Fig 6:**
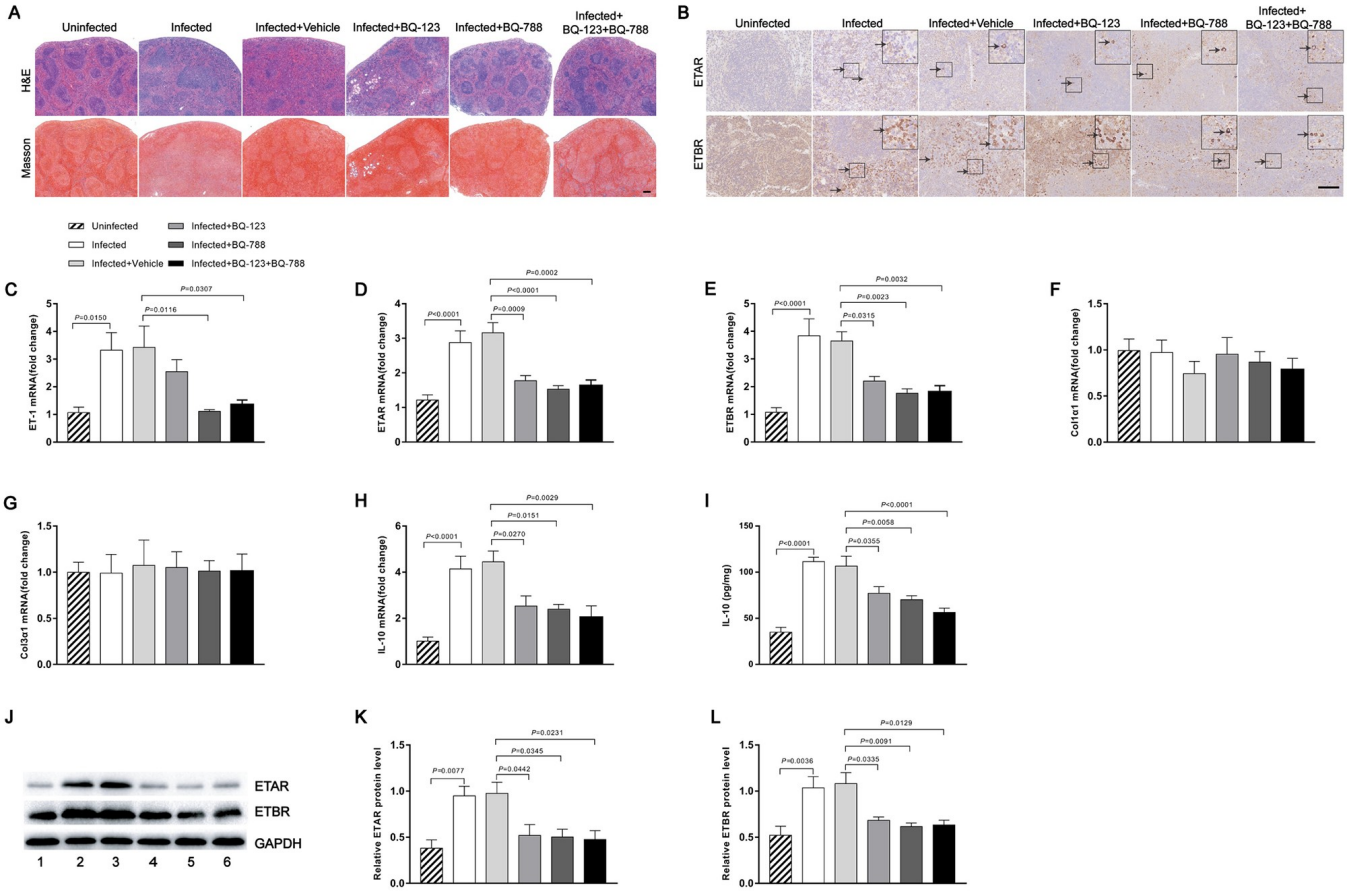
Endothelin receptor antagonists attenuated schistosomiasis-induced splenic fibrosis in mice. (A) H&E and Masson’s trichrome staining of spleen sections. Scale bar, 200 μm. (B) Representative immunohistochemical staining for ETAR and ETBR in infected spleens. *Insets* show a higher magnification of the outlined area. Black arrows indicate the ETRs positive cells. Scale bar, 100 μm. (C-H) The expression levels of ET-1, ETAR, ETBR, Col1α1, Col3α1 and IL-10 in spleens were determined by qPCR (n = 7). (I) The IL-10 concentration in spleen tissue homogenates was determined by ELISA (n = 6). (J-L) ETAR and ETBR proteins were determined by western blotting. 1, uninfected; 2, infected; 3, infected + vehicle; 4, infected + BQ-123; 5, infected + BQ-788; 6, infected + BQ-123 + BQ-788. Image density was quantified using Image J analysis and normalized to GAPDH (n = 5). Data are represented as mean ± SEM of three independent experiments. Multiple comparisons were performed by one-way ANOVA with Tukey’s correction for comparison between two groups (C-I, K-L).

Next, we evaluated the levels of splenic fibrosis in these mice. Consistent with the attenuated fibrotic phenotype, a reduction of structural damage was detected in spleens of mice treated with endothelin receptor antagonists as shown by H&E staining, while collagen deposition was not significantly altered in these mice by Masson’s trichrome staining (Fig 6A). The protein levels of ETAR and ETBR were decreased in the spleen tissues of mice treated with endothelin receptor antagonists compared with vehicle tissues, as determined by immunohistochemistry and western blotting (Fig 6B and 6J–6L). The expression levels of ETAR, ETBR, and IL-10 mRNA in the treated spleen tissues were down-regulated (Fig 6D, 6E and 6H). The ET-1 mRNA expression level was decreased in the presence of BQ-788 and BQ-123 + BQ-788. Although the BQ-123-treated group appeared to exhibit a slight reduction ET-1 mRNA compared with the vehicle group, the difference was not statistically significant (Fig 6C). As expected, the levels of Col1α1 and Col3α1 mRNA were unchanged among all study groups (Fig 6F and 6G). In addition, the IL-10 concentration in spleen tissue homogenates, as detected by ELISA, was decreased in the treated groups compared with the vehicle group (0.5 to 0.7-fold) (Fig 6I). Taken together, these results suggest that endothelin receptor antagonists can partially reverse schistosome-induced hepatic fibrosis.

### Endothelin receptor antagonist-mediated down-regulation of IL-10 expression in splenic B cells

**Fig 7:**
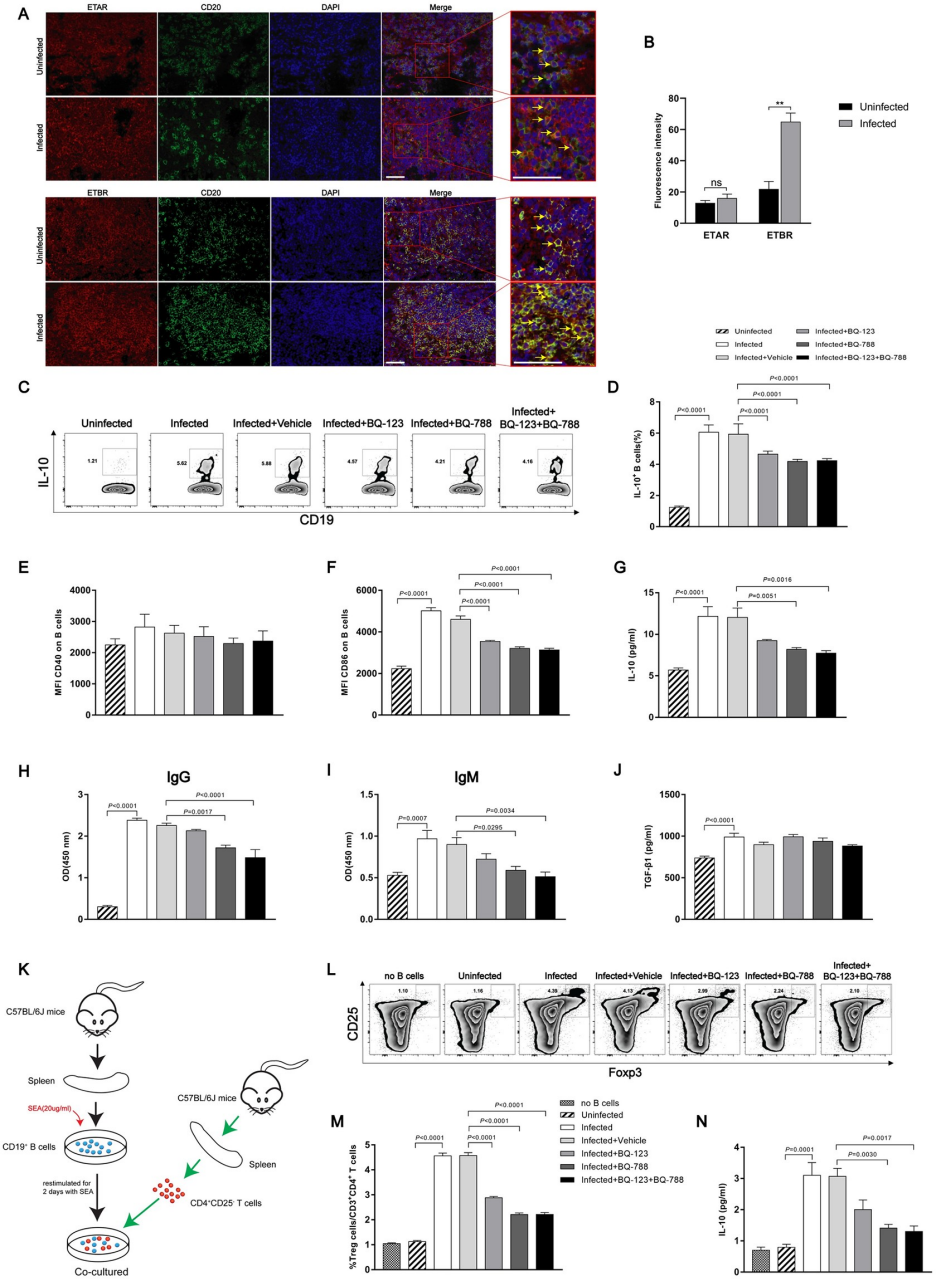
Endothelin receptor antagonists inhibited B cell activation. (A) Representative immunofluorescence staining of ETAR, ETBR and CD20^+^ B cells in mouse spleen tissues. *Insets* show a higher magnification of the outlined area. Yellow arrows denote positive cells. Scale bar, 50 μm. (B) The fluorescence intensity was measured using IPP software (n = 4). (C-J) CD19^+^ MACS-isolated mouse splenic B cells were restimulated with SEA (20 μg/ml) for 2 days. (C-D) Representative FACS plots and a summary of the intracellular IL-10 expression of B cells after addition of Brefeldin A to the last 4 hours of the culture (n = 7). (E-F) Mean fluorescence intensity of CD40 and CD86 expression (n = 7). (G-J) IL-10, IgG, IgM and TGF-β1 concentration in culture supernatants as determined by ELISA (n = 6–8). (K-N) SEA-restimulated B cells were co-cultured for 4 days with CD25^-^ depleted CD4 T cells. (L-M) The frequency of CD25^+^Foxp3^+^ Treg cells after co-culture, as shown in a representative FACS plots and summarized (n = 7). (N) IL-10 concentration in culture supernatants after co-culture (n = 3). Data are represented as mean ± SEM of three independent experiments. Significance was calculated using the two-tailed Student’s t test (B) or one-way ANOVA with Tukey’s correction for comparison between two groups (D-J, M-N). ns, not significant; **P< 0.01.

To further evaluate the role of ETRs in murine schistosome-induced hepatic fibrosis, we then investigated the presence of ETAR and ETBR in spleen tissues. We found that B cells in mice spleen tissues expressed ETAR and ETBR in vivo based on immunofluorescence staining (Fig 7A). Furthermore, the fluorescence intensity of ETBR (3.0-fold), but not ETAR, was increased in infected compared with uninfected spleens (Fig 7B). There was a patchy distribution of ET receptors in splenic B cells. In contrast, ETRs were scarce on endothelial cells of the splenic sinus (S8 Fig). Elevated IL-10 characterizes chronic stages of schistosome infection and is produced by B cells starting from week 8 during infection (1.4 to 2.5-fold) (S10 Fig). Previous studies have identified B cells as the major IL-10-producing cells during chronic schistosome infection and mediating protection in a mouse model of airway inflammation [36]. Furthermore, SEA-stimulated splenic mononuclear cells from mice depleted of B cells exhibited lower levels of IL-10 in culture supernatants compared to control mice, suggesting that IL-10-producing B cells may play an important role in the process of schistosomiasis infection (0.7-fold) (S11A–S11C Fig). To elucidate the mechanism by which *S*. *japonicum* can drive the development of B cells, we isolated splenic B cells from C57BL/6J mice in the different groups as described above. The frequencies of B cells expressing intracellular IL-10 protein in the endothelin receptor antagonist-treated mice were reduced compared with the vehicle group (0.7 to 0.8-fold) (Fig 7C and 7D). Additionally, the surface activation marker CD86, which is often upregulated on activated B cells [37, 38], was decreased on splenic B cells in response to endothelin receptor antagonist treatment (Fig 7F), while CD40 expression was not significantly changed (Fig 7E). Splenic B cells from the BQ-788 and BQ-123 + BQ-788-treated mice showed significantly decreased IL-10 secretion (approximately 0.6-fold), as measured in culture supernatants. While the BQ-123-treated group was not significantly changed (approximately 0.8-fold) (Fig 7G). We further investigated the ability of endothelin receptor antagonists to affect B cell responses. The culture supernatant of splenic B cells was also collected and the levels of IgG and IgM were detected with ELISA. Compared with the vehicle group, BQ-788 and BQ-123 + BQ-788-treated groups significantly decreased the ability of B cells to secrete IgG and IgM antibodies (0.6 to 0.7-fold). While the BQ-123-treated group was not significantly changed (0.9-fold) (Fig 7H and 7I). We did not find a significant difference in the secretion of TGF-β1 from splenic B cells (Fig 7J). To confirm the regulatory function, splenic B cells from the various groups were tested for their capacity to drive Treg cell development, an acquired phenotype that has previously been described for splenic B cells during natural schistosome infection. Indeed, splenic B cells from endothelin receptor antagonist-treated, but not vehicle group, mice showed decreased development of CD25^+^ Foxp3^+^ Treg cells during 4 days of co-culture with CD25^-^ depleted CD4 T cells (0.5 to 0.6-fold) (Fig 7K–7M). In addition, the IL-10 protein concentration in the co-culture supernatants was decreased in the presence of BQ-788 and BQ-123 + BQ-788 (approximately 0.4-fold). Although the BQ-123-treated group appeared to display a slight reduction of IL-10 expression compared with the vehicle group, the difference was not statistically significant (0.7-fold) (Fig 7N). Collectively, these data demonstrate that endothelin receptor antagonists can mediate the down-regulation of IL-10 expression in splenic B cells.

**Fig 8:**
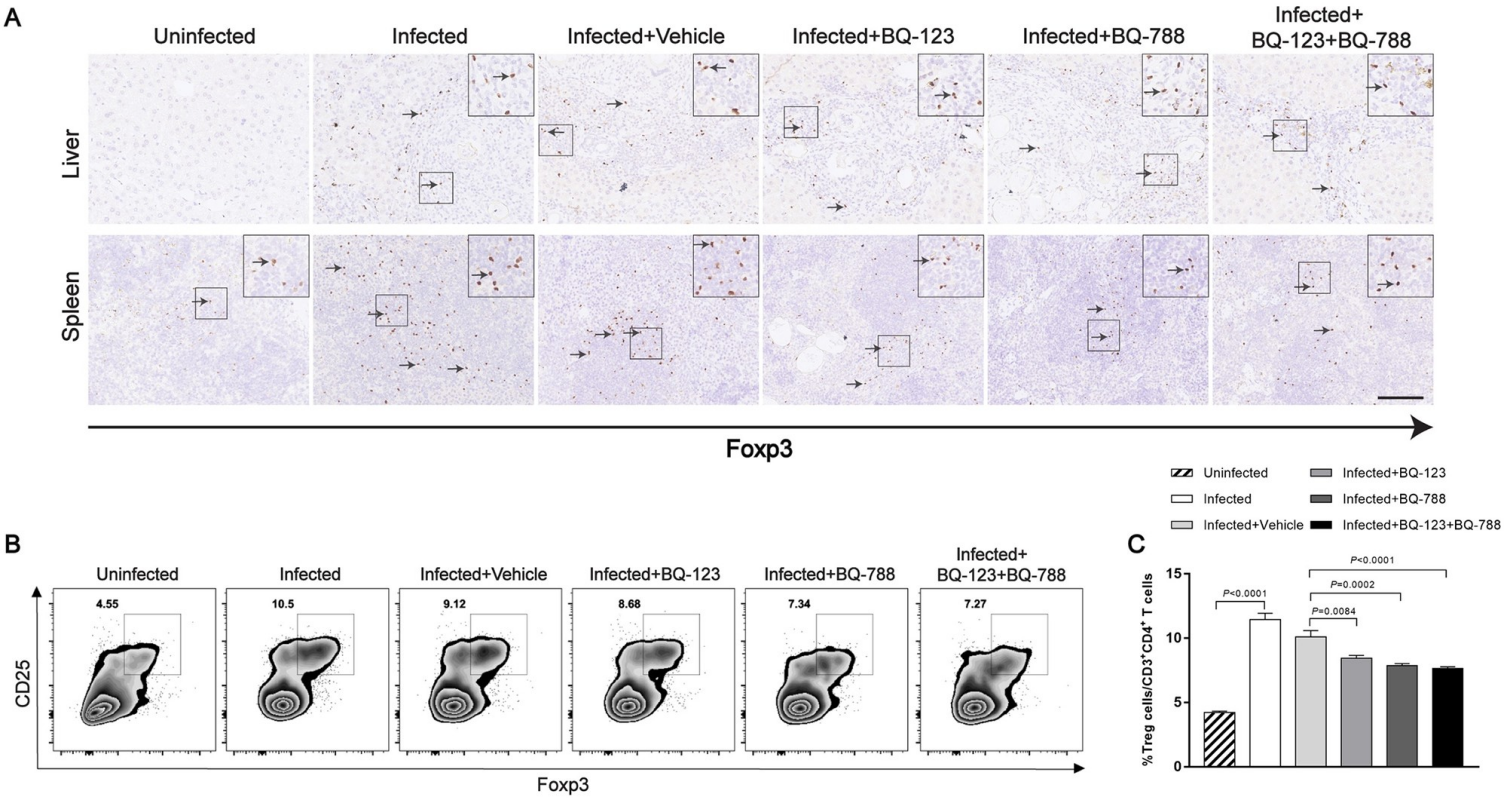
Endothelin receptor antagonists decreased CD4^+^CD25^+^Foxp3^+^ T cells in mouse spleen tissues. (A) Representative immunohistochemical staining for Foxp3 in infected livers and spleens. *Insets* show a higher magnification of the outlined area. Black arrows indicate the Foxp3^+^ positive cells. Scale bar, 100 μm. (B-C) The frequency of CD25^+^Foxp3^+^ Treg cells in mouse splenocytes, as shown in a representative FACS plots and summarized (n = 7). Data are represented as mean ± SEM of three independent experiments. Multiple comparisons were performed by one-way ANOVA with Tukey’s correction for comparison between two groups (C).

Our data have shown that endothelin receptor antagonists have the ability to prevent the development of splenic B cells characterized the Treg cell-inducing capacity. To better understand that B cell activation in vivo is reduced in the presence of antagonists, immunohistochemistry was conducted to determine the Foxp3 expression in liver and spleen tissues. Representative photomicrographs revealed that enhanced Foxp3 in liver and spleen tissues from mice infected with *S*. *japonicum* and Foxp3-positive cells were localized mainly in areas of inflammation and fibrosis of the liver (Fig 8A). We also found that the Foxp3 expression was decreased in spleen but not in liver of mice treated with endothelin receptor antagonists (Fig 8A). Flow cytometry was performed to examine the frequency of CD4^+^ CD25^+^ Foxp3^+^ Tregs in mouse splenocytes. Our results showed that the frequency of splenic Tregs was decreased after treated with endothelin receptor antagonists (approximately 0.8-fold) (Fig 8B and 8C). These data demonstrate that endothelin receptor antagonists are capable to suppress the activation of splenic B cells both in vivo and in vitro.

## Discussion

It is well known that ETRs have an important function in non-infectious liver disease. However, the role of ETRs in parasitic infection has remained largely unclear. In the current study, we provide evidence for a functional role of ETRs in human and murine hepatic fibrosis with *S*. *japonicum*. Our data indicate that the up-regulation of ETAR and ETBR is crucial for the progression of hepatic fibrosis induced by schistosome infection, and ETRs are predominantly expressed on splenic B cells in spleen tissues. Importantly, our study suggests that endothelin receptor antagonists can partially reverse schistosome-induced hepatic fibrosis by suppressing the activation of splenic B cells during infection.

Endothelin receptor expression is upregulated in liver disease, and HSCs express the highest levels of ETRs [23, 39]. Furthermore, endothelin-induced contraction is enhanced in stellate cells from cirrhotic rat livers, and endothelin causes sustained vasoconstriction in the intact liver [40]. For more than a decade, there has been intense interest in the possible role of ETRs in the pathogenesis of liver fibrosis and cirrhosis [41, 42]. However, no study to date has examined the role of ETRs in hepatic fibrosis caused by schistosomiasis. In this study, we examined the expression of fibrosis markers in control patients with normal liver and with hepatic fibrosis. Our results showed that the expression levels of ETAR and ETBR were significantly higher in liver tissues with hepatic fibrosis, and their expression also appeared to be linked to the histological degree of schistosomiasis hepatic fibrosis; this outcome provided evidence of their involvement in the progression of schistosomiasis.

Advanced schistosomiasis is typically characterized by splenomegaly, portal hypertension, and bleeding esophageal varices [43]. Splenic enlargement is one of the most palpable abnormalities accompanying liver cirrhosis, and it frequently occurs in parallel with hypersplenism [44]. In this work, we observed high levels of ETAR and ETBR in the spleen tissues of patients with CS or CHB, and the levels were slightly higher in CS patients. The most important histological alterations in the spleen of CS and CHB patients were blood congestion in the red pulp and increases in the number of spleen macrophages, with hyperplasia of increased reticular fibers and of subcapsular myo-fibroblasts. In addition, we broadened the focus of our study by providing indirect evidence of the major roles played by the two molecules by analyzing the correlations between their mRNA expression and certain parameters. The mRNA levels of ETAR and ETBR in the spleen tissues of CS patients had a relationship with spleen thickness diameter and portal vein diameter, which were increased in parallel with the extent of hepatic fibrosis [45, 46]. More importantly, we showed that endothelin receptor-producing cells were mainly located in splenic B cells and scantily in endothelial cells of the splenic sinus. We further found that the expression of ETAR and ETBR in B cells was significantly elevated after infection. These studies suggest that ETAR and ETBR might be involved in the pathogenesis of liver fibrosis via splenic B cells during schistosome infection.

To further confirm the roles of ETAR and ETBR in vivo, we established a murine model of *S*. *japonicum* infection based on information from previous studies [47, 48]. Our study indicated that schistosome-infected mice showed higher ETAR and ETBR expression than the uninfected group both in liver and spleen, and they also showed an association with advanced stages of liver fibrosis. However, collagen deposition was not altered in the spleen of the mice; this outcome is inconsistent with the results obtained for human spleen. Based on the histology of the spleen, we found that *S*. *japonicum* infection destroyed the structure of the lymphoid follicles in the spleen of mice, while the spleen pathology of schistosomiasis patients was a main feature of fibrogenesis. This inconsistency might be due to the differences in harvest time. As lymphoid follicles are gradually disrupted, residual lymphocytes and other immune cells could contribute to the development of the spleen pathology [49]. Differences in heterogeneity may also explain the disagreement, as even the mice treated under the same infection conditions showed different spleen pathologies [50].

Numerous studies have suggested that endothelin receptor antagonists have antifibrotic activity [51]. ET-1 is chemotactic for cells of the fibroblast lineage and causes fibroblast proliferation and ECM accumulation and contraction, mediated by ETAR and ETBR expressed on these cells [52]. Thus, antagonists of both ET-1 receptors may have therapeutic potential to prevent the development of fibrosis. This concept has been supported by studies of animal models with portal hypertension, and these studies have shown that administration of endothelin receptor antagonists reduces portal pressure [53]. We illustrated a significant contribution of both ETA and ETB receptors during the progression of hepatic schistosomiasis. These results were confirmed by the use of the selective ETA or ETB antagonists BQ-123 and BQ-788, both of which caused a significant reduction of hepatocellular damage and partially reversed schistosome-induced hepatic fibrosis. Curiously, selective blockade of ETB receptors with BQ-788 showed more apparent affinity in the response to infection. Probably due to the patchy distribution of ET receptors in splenic B cells, BQ-788 showed stronger inhibition of splenic B cells characterized by IL-10 and antibodies secretion and Treg cell-inducing capacity. Our finding that B cells in mouse spleen tissues expressed ETAR and ETBR in vivo raises the question of whether those receptors can directly interact with B cells during schistosome infection. Splenic B cells are the most prominent source of IL-10 [36]. The role of splenic B cells in schistosomiasis has also been extensively studied. Haeberlein S et al. reported that, in an animal model of *S*. *mansoni* infection, antigens directly interact with splenic B cells of mice, triggering them to produce the anti-inflammatory cytokine IL-10 and increasing their capacity to induce Treg cells [54]. This phenomenon has also been observed in an animal model of *S*. *japonicum* infection [55, 56]. In the present study, our data also indicated that IL-10 produced by B cells was elevated during the progression of schistosome infection. We found that endothelin receptor-producing cells were mainly located in splenic B cells, and the expression of ETRs in splenic B cells was upregulated during schistosome infection. Furthermore, we demonstrated that blocking ETRs on B cells from infected mice suppressed the activation of splenic B cells both in vivo and in vitro, and the fibrosis was attenuated after treated with endothelin receptor antagonists, suggesting the endothelin receptor antagonists had a beneficial effect on schistosome-induced hepatic fibrosis through preventing the development of splenic B cells characterized by IL-10 secretion and the Treg cell-inducing capacity.

As illustrated above, evidence from a few human and animal studies points towards a significant role for ETRs in modulating pathogenic parasitic responses. Our data shed new light on a possible common paradigm regarding how ETAR and ETBR regulate fibrosis in different organs, and strongly suggest that ETRs aggravate liver fibrosis caused by *S*. *japonicum* via splenic B cells. Although praziquantel has been shown to be an effective anthelmintic drug for destroying the parasite in schistosomiasis, there is no highly effective approach to mitigate hepatic fibrosis caused by schistosomiasis [13]. In past decades, splenectomy has been utilized to ameliorate the fatal complications of fibrosis-associated portal hypertension. However, many fibrosis patients present with contraindications that preclude splenectomy. Thus, it remains critical for us to identify alternative novel and non-surgical methods for the treatment of liver fibrosis. Our data clearly illustrate that endothelin receptor antagonists alleviate such a hepatic fibrosis by suppressing the activation of splenic B cells. However, further clinical and experimental studies are necessary to evaluate the specificity and safety of the pharmacological treatment of these human diseases.

## Materials and methods

### Ethics statement

The human study was approved by the Ethics Committee of Tongji Hospital, Tongji Medical College, Huazhong University of Science and Technology (Permit Number: 20150103). Studies were performed according to the declaration of Helsinki and all participants were adults and have given written informed consent. All animal experiments were performed in strict accordance with the Guide for the Care and Use of Laboratory Animals of the National Institutes of Health, and were approved by the Institutional Animal Care and Use Committee of Tongji Medical College, Huazhong University of Science and Technology (IACUC Number: 629). To minimize pain and discomfort, all animal surgeries were anesthetized with sodium pentobarbital.

### Patients

All samples were obtained from Tongji Hospital from September 2015 to August 2019. Liver specimens were collected from 46 patients with hepatic fibrosis or cirrhosis, including 31 patients with CS and 15 patients with CHB. As Cont, wedge biopsy specimens from normal portions of the liver were obtained from 8 patients with metastatic liver carcinoma (3 colonic carcinomas and 5 gastric carcinomas). Liver fibrosis (Stage) of all liver biopsies was assessed according to the Scheuer scoring system [57]. Cirrhotic spleen specimens were obtained from 25 patients which contained 12 CS patients and 13 CHB patients who required splenectomy, and 9 patients who underwent splenectomy for trauma as Cont. All patients with CS had a history of infected water exposure and praziquantel treatment. Patients with CHB were positive for hepatitis B virus surface antigen for over 6 months. Study exclusion criteria included patients with other types of hepatitis and liver disease associated with drugs or alcohol. No patient had either pulmonary or cardiac disease. Unless otherwise stated, n-values refer to the number of patients from whom tissue was obtained. Because of the limited amount of tissue samples, not all samples were included in every study protocol. All patients were subjected to complete history taking, thorough clinical examinations, routine laboratory investigation, and abdominal ultrasonography. The demographic and clinical characteristics of the patients are summarized in Table 1.

**Table 1:** Patients’ demographic characteristics.

	Cont	CS	CHB	*P* value
Liver samples				
Subject no.	8	31	15	
Male sex	5(63%)	19(61%)	11(73%)	0.7170
Age(y)	62(59–66)	50(43–62)	52(48–55)	0.0543
History of *S*. *japonicum*/hepatitis B virus infection				
<10 years	0(0%)	2(7%)	3(20%)	
10–20 years	0(0%)	14(45%)	4(27%)	
>20 years	0(0%)	15(48%)	8(53%)	
Stage of fibrosis				
S0	8(100%)	0(0%)	0(0%)	
S1	0(0%)	6(19%)	0(0%)	
S2	0(0%)	5(16%)	0(0%)	
S3	0(0%)	8(26%)	0(0%)	
S4	0(0%)	12(39%)	15(100%)	
Splenectomy(S4%)	0(0%)	9(75%)	8(53%)	**0.0040**
Ascites(S4%)	0(0%)	5(42%)	4(27%)	0.1120
EGVB(S4%)	0(0%)	4(33%)	6(40%)	0.1170
Spleen samples				
Subject no.	9	12	13	
Male sex	5(56%)	7(58%)	7(54%)	0.9750
Age(y)	53(52–64)	51(43–65)	51(45–56)	0.3397
History of *S*. *japonicum*/hepatitis B virus infection				
<10 years	0(0%)	0(0%)	4(31%)	
10–20 years	0(0%)	5(42%)	6(46%)	
>20 years	0(0%)	7(58%)	3(23%)	
Ascites	4(44%)	5(42%)	3(23%)	0.4980
EGVB	0(0%)	6(50%)	3(23%)	**0.0350**

For continuous variables, results are expressed as median and interquartile range. Categorical variables are summarized by using percentages. Boldface indicates *P* <0.05. S4%, the percentage of S4 population; Cont, control; CS, chronic schistosomiasis; CHB, chronic hepatitis B; EGVB: esophagealgastricvariceal bleeding

### Mice, infection and antigen preparation

Female C57BL/6J mice (6 week) were purchased from Beijing Vital River Laboratory Animal Technology Co., Ltd. (Beijing, China) and housed under specific pathogen-free conditions with standard laboratory food and water ad libitum. To establish the animal model of schistosomiasis, mice were exposed percutaneously to 16 ± 1 *S*. *japonicum* cercariae (Chinese mainland strain) obtained from infected Oncomelania hupensis snails purchased from Nanjing Institute of Schistosomiasis Prevention and Control (Nanjing, China). The mice were sacrificed at 4, 6, 8 and 12 weeks post-infection, and specimens, including liver and spleen tissues and serum, were collected. Soluble egg antigens (SEA) was obtained from purified and homogenized *S*. *japonicum* eggs. The protein concentration of SEA was determined using a bicinchoninic acid (BCA) assay (Pierce, Cramlington, UK).

### Treatment of hepatic fibrosis

BQ-123 (HY-12378, MedChemExpress) and BQ-788 (HY-15894, MedChemExpress) were dissolved in dimethyl sulfoxide (DMSO) according to the manufacturer’s instructions and then diluted in PBS to a final bath concentration of 1% DMSO. After 6 weeks of C57 mouse infection with cercariae, Vehicle (1% DMSO), BQ-123, BQ-788, and BQ-123 plus BQ-788 were intraperitoneally (i.p.) administered to mice at doses of 50 μg/kg three times a week for 6 weeks. Then, mice were harvested for further analysis.

### Parasite perfusion, and egg counting

For parasite perfusion, the portal vein was dissected at the root. The circulatory system was perfused via the aorta with sterile PBS, and worms were collected and counted in a sterile petri dish containing medium, as described previously [58]. The number of schistosome eggs in the liver was counted after part of the liver was digested overnight with 4% potassium hydroxide, and the liver egg burdens were expressed as 10^4^ eggs per gram of liver tissue.

### Liver histopathology and fibrosis measurement

Liver or spleen index was calculated according to the following formula: (liver or spleen weight/body weight) ×100. The size of the egg granuloma was measured from by Mayer’s H&E staining of sections using a calibrated measuring eyepiece, and the extent of fibrosis was evaluated by Masson’s trichrome staining of sections as described previously [59]. The total fibrosis score was determined by multiplying the density and area for each granuloma (a score of 16 would be the maximum). The collagen content of the liver, determined as hydroxyproline content, was detected using a colorimetric assay according to the manufacturer’s instructions (Nanjing Jiancheng Bioengineering Institute, Nanjing, China).

### Serum biochemistry

Serum levels of alanine aminotransferase (ALT) and aspartate aminotransferase (AST) were measured using a Siemens Advia 1650 automatic analyzer.

### Liver portal vein diameter and pressure

The mice were fasted overnight after the last injection, and they were anesthetized with sodium pentobarbital (60 mg/kg, i.p.) and dissected to reveal the portal vein. Portal vein diameters were determined using digital calipers at three points in vivo: first, at the emergence from the pancreas; second, inferior to the porta hepatis; third, halfway between the first two points. For each portal vein, three measurements were averaged to represent a mean venous diameter. The portal vein was cannulated with a PE-50 catheter (American Health & Medical Supply International Corp, USA), and the pressure was recorded with the Multi-Channel Physiological Signal Collecting and Processing System (RM6240BD, Chengdu Instrument Factory, China).

### Immunohistochemistry and immunofluorescence

Paraffin-embedded sections (4 mm) were deparaffinized and rehydrated and then subjected to heat-induced antigen retrieval using Target Retrieval Solution (Dako, Carpinteria, Calif). The 3% hydrogen peroxidase was used to inhibit endogenous peroxidase activity, and 5% BSA was used to block nonspecific antibody binding. For immunohistochemistry, horseradish peroxidase (HRP)-conjugated secondary antibodies (Pepro Tech Inc., USA) were used for immunostaining with the following primary antibodies: rabbit polyclonal antibody against ETAR (1:2000, ab117521, Abcam, Cambridge, MA), rabbit polyclonal antibody against ETBR (1:2000, ab117529, Abcam, Cambridge, MA), rabbit polyclonal antibody against collagen I (1:100, 14695-1-AP, ProteinTech, Chicago, USA), rabbit polyclonal antibody against collagen III (1:250, 22734-1-AP, ProteinTech, Chicago, USA) and rabbit monoclonal antibody against Foxp3 (1:50, 12653, CST, USA). Color development was achieved with 39, 39-diaminobenzidine. Representative photomicrographs of tissue sections stained with control antibodies are shown in S12 Fig. For immunofluorescence staining, tissue sections were incubated with the primary antibody against ETAR (1:5000, ab117521, Abcam, Cambridge, MA), ETBR (1:5000, ab117529, Abcam, Cambridge, MA), CD20 (1:100, ab64088, Abcam, Cambridge, MA) and CD31(1:20, ab28364, Abcam, Cambridge, MA), followed by Alexa Fluor 594- and Alexa Fluor 488-conjugated secondary antibodies (1:200, Invitrogen, Carlsbad, CA). Finally, all the sections were stained with 1 μg/ml 4′,6′-diamidino-2-phenylindole (DAPI; Sigma-Aldrich) to visualize cell nuclei. At least three liver sections were included in each group. The stained sections were viewed under a microscope(NIKON Eclipse Ci), and images were captured using a high-resolution digital camera (NIKON digital sight DS-FI2) and analyzed with Image-Pro Plus 6.0 software (IPP, Media Cybernetics, Bethesda, MA, USA).

### RNA analysis

Total RNA was isolated using TRIzol reagent (Invitrogen) according to the manufacturer’s protocol. Real-time quantitative polymerase chain reaction (qPCR) was performed as described previously [60]. The expression levels of ET-1, ETAR, ETBR, Col1α1, Col3α1 and IL-10 were determined using the SYBR Green Master Mix kit (Takara, Kusatsu, Japan). GAPDH was used as an internal control, and the fold change was calculated by the 2^-ΔΔCt^ method. The human and mouse primer sequences are described in Table 2.

**Table 2:** Primer sequences used in this study.

Gene	Species	Primer sequence (5'-3')	Size (bp)
ET-1	Human	F: GAGAATTTACTTCCCACAAAGGC R: TCCATAATGTCTTCAGCCCTG	125
ETAR	Human	F: TGCCCTCAGTGAACATCTTAAG R: CATCGGTTCTTGTCCATCTCG	147
ETBR	Human	F: CAAGGACCCATCGAGATCAAG R: AGATATTGGGACCGTTTCGC	145
Col 1α1	Human	F: GAAGACATCCCACCAATCACC R: TCTCGTCACAGATCACGTCATC	136
Col 3α1	Human	F: CTACTTCTCGCTCTGCTTCATC R: CACAGACACATATTTGGCATGG	136
IL-10	Human	F: GACAGACTTGCAAAAGAAGGC R: TCTCGAAGCATGTTAGGCAG	149
GAPDH	Human	F: GGAGCGAGATCCCTCCAAAAT R: GGCTGTTGTCATACTTCTCATGG	197
ET-1	Mouse	F: TTCTTGCCGGTTGGGAATGA R: TTTCTACAGAAACCCCGCCC	118
ETAR	Mouse	F: GTCTTGAACCTCTGTGCTCTC R: GATCCCGATTCCTTGAACTCG	78
ETBR	Mouse	F: TGAGTATTGACAGATATCGAGCTG R: AAAACCTATGGCTTCAGGGAC	137
Col 1α1	Mouse	F: CGCCATCAAGGTCTACTGC R: ACGGGAATCCATCGGTCA	152
Col 3α1	Mouse	F: GCCCACAGCCTTCTACACCT R: GCCAGGGTCACCATTTCTC	110
IL-10	Mouse	F: CAACATACTGCTAACCGACTCCT R: TGAGGGTCTTCAGCTTCTCAC	173
GAPDH	Mouse	F: CCTCGTCCCGTAGACAAAATG R: TGTAGTTGAGGTCAATGAAGGG	139

### Western blotting

Total cell protein was extracted on ice with RIPA lysis buffer (Beyotime biotechnology, Shanghai, China) in the presence of freshly added protease inhibitors (Boster Biological Technology, Wuhan, China), and quantified by the BCA assay (Pierce, Cramlington, UK). A total of 30 μg protein from each sample was subjected to 10% SDS–PAGE (Beyotime biotechnology, Shanghai, China) by electrophoresis under reducing conditions and transferred to PVDF membrane (Millipore Corporation, Billerica, MA, USA). The blotted membrane was then blocked with 5% skim milk for 1 h at room temperature and incubated respectively with anti-ETAR (1:5000, ab117521, Abcam, Cambridge, MA), ETBR (1:5000, ab117529, Abcam, Cambridge, MA) and anti-GAPDH (1:2000, 10494-1-AP, ProteinTech, Chicago, USA) antibody overnight at 4°C. The membranes were further incubated with HRP-conjugated anti-rabbit secondary antibodies and detected using the enhanced chemiluminescence (ECL; Abbkine, Redlands, CA, USA) method. The densitometry was performed using Image J software.

### Measurement of SEA-specific IgG

Levels of SEA-specific anti-IgG in serum of infected mice were measured with an ELISA kit (Shenzhen Combined Biotech Co. Ltd., China). All serum samples were diluted 1:100 and transferred into the kit supplied microtiter wells. The incubation procedure, washing steps and detection steps were carried out according to the manufacturer’s instructions. Optical density (OD) values were read at 450 nm zeroed by the reagent blank wells. For each run, positive and negative control sera were measured simultaneously. A positive result was defined as an OD value greater than 2.1 times the OD value of the negative control serum provided by the kit, as specified by the manufacturer’s instructions.

### Cytometric bead array (CBA)

The concentration of IFN-γ, TNF, IL-2, IL-4, IL-5 and IL-13 in serum from mice was measured with the use of cytometric bead array kits (BD Biosciences, San Diego, CA) according to the manufacturer’s recommendations. The data were analyzed by FCAP Array V3 Software.

### Isolation of splenocytes, B cells and T cells

Single cell suspensions of murine spleens were prepared by passing cells through a 70-μm cell strainer (BD Biosciences, Breda, The Netherlands), and erythrocytes were depleted by lysis. B cells were purified from spleens by using anti-CD19 MicroBeads (Miltenyi Biotec, Bergisch Gladbach, Germany), following the manufacturer’s protocol. The purity was greater than 97%, and the viability was greater than 95%. CD4^+^CD25^-^ T cells were enriched using anti-CD4 and anti-CD25 MicroBeads with a purity of 96% (Miltenyi Biotec). Representative results for the purification of B cells and CD4^+^CD25^-^ T cells are shown in S9 Fig.

### In vitro murine B cell stimulation and co-culture with CD4^+^CD25^-^ T cells

Mouse splenic CD19^+^ B cells (1.5x10^6^/ml) were cultured in medium (RPMI 1640 glutamax; Thermo Fisher Scientific) containing 10% heat-inactivated fetal bovine serum (FBS; Gibco, Gaithersburg, MD, USA) and 100 μg/mL penicillin/streptomycin (Invitrogen, Carlsbad, CA, USA) and restimulated for 2 days with SEA (20 μg/ml) to allow the detection of cytokines, as previously established for in vivo schistosome-exposed B cells [36]. Supernatants were collected for cytokine analysis by ELISA. Cells were cultured for an additional 4 hours with Brefeldin A (10 μg/ml; MedChemExpress) to detect intracellular IL-10 by flow cytometry. For in vitro Treg cell induction, SEA-stimulated CD19^+^ B cells were co-cultured with MACS-sorted CD4^+^CD25^-^ T cells at a 1:1 ratio (1 x 10^6^/ml each). After 4 days, the frequencies of Treg cells were determined by flow cytometry by gating on Foxp3^+^CD25^+^ cells in the CD3^+^CD4^+^ T cell population, and culture supernatants were collected for subsequent cytokine determination.

### B cell depletion and in vitro experiments

Mice were injected intravenous with 250 μg of anti-mouse CD20 (clone SA271G2, BioLegend) or with control isotype. Depletion was confirmed by staining of splenic cells with anti-mouse CD19 (BD Biosciences) at day 7. Splenic mononuclear cells were obtained by Ficoll-Hypaque gradients and were cultured in the presence of SEA (20 μg/ml) for 3 days. Supernatants were stored for IL-10 analysis by ELISA.

### Flow cytometry

Mononuclear cells were blocked with anti-mouse CD16/CD32 (Fc block; Biolegend) prior to antibody staining. All cells were first stained with the Fixable Viability Stain 700 (BD Biosciences) as a live/dead discriminator [61]. Flow cytometric analysis of murine B cells was detected by staining with fluorochrome-labeled antibodies against CD19 (BD Biosciences), B220, CD40, and CD86 (all Biolegend). For intracellular staining of IL-10 (Biolegend), after surface staining, cells were fixed and permeabilized with the Intracellular Fixation&Permeabilization Buffer Set (eBioscience). Treg cells were fixed and permeabilized with the Foxp3/Transcription Factor Staining Buffer Set (eBioscience) and stained using fluorochrome-labeled antibodies against CD3, CD4, CD25 and Foxp3 (all Biolegend). Flow cytometry was performed using a BD LSRFortessa (BD Biosciences), and flow cytometric data were analyzed with FlowJo software (Version 10.0, Tree Star, Inc).

### ELISA

Tissue homogenates were generated as previously described [62]. Culture supernatants were concentrated ∼5-fold using an Amicon Ultra-10 centrifugal filter device (Millipore, USA) according to the manufacturer’s protocol. The concentrations of IL-10 and TGF-β1 were quantified using commercial ELISA kits according to the manufacturer’s instructions (Boster Biological Technology, Wuhan, China). The levels of IgG and IgM in culture supernatants were detected using ELISA kits following the manufacturer’s instructions (Solarbio, Beijing, China).

### Statistical analysis

The results of the experimental data are expressed as the mean ± standard error of the mean (SEM). Statistical significance between experimental groups was assessed using the two-tailed Student’s t test and one-way ANOVA with Tukey’s correction. The results of the clinical data from patients are expressed as the median and interquartile range, due to the skewed distributions of most variables in patients, and the Mann-Whitney U-test and Kruskal–Wallis with Dunn’s multiple comparisons posttest for data concerning clinical results. For categorical data, χ^2^ or Fisher exact tests were performed to determine the difference between groups. Spearman’s rank test was used for correlations. All statistical analyses were performed with GraphPad Prism 7.0 software (GraphPad Software, La Jolla, CA, USA) or SPSS 25.0 software (SPSS, Chicago, Ill). *P* values < 0.05 were considered significant.

## Supporting information

S1 Fig Histological findings for ETRs in liver tissues from patients infected with *S*. *japonicum*. (A) Evaluation of the fibrotic stage in liver biopsy specimens by H&E and Masson’s trichrome staining. Paraffin-embedded sections of liver tissues from patients were stained with H&E, Masson’s trichrome and Sirius Red. Black arrows indicate worm eggs. Scale bar, 200 μm. (B) The positive staining areas for Sirius Red were measured using IPP software (n = 4). (C) Representative immunohistochemical staining of ETAR and ETBR. Black arrows indicate the ETRs positive cells. Scale bar, 100 μm. Data are represented as the median and interquartile range of two independent experiments. Significance was determined by the Mann-Whitney U-test. *P < 0.05, compared with S0 samples.
(TIF)

S2 Fig ETRs were scantily located in the endothelial cells of the splenic sinus. Representative immunofluorescence staining of ETAR, ETBR and CD31^+^ endothelial cells in human spleen tissues. Yellow arrows denote positive cells. Scale bar, 100 μm.
(TIF)

S3 Fig Examination of clinical indicators. (A-F) ALT, AST, TBIL, ALP, GGT and PLT in patients who underwent splenectomy. Cont (n = 9), CS (n = 12), CHB (n = 13). Data are represented as the median and interquartile range. Significance was calculated using Kruskal–Wallis with Dunn’s posttest. Abbreviation: Cont, control; CS, chronic schistosomiasis; CHB, chronic hepatitis B; ALT: alanine aminotransferase; AST: aspartate aminotransferase; TBIL: total bilirubin; ALP: alkaline phosphatase; GGT: gamma-glutamyl transpeptidase; PLT: platelet.
(TIF)

S4 Fig Additional data for the analysis in murine schistosome-induced hepatic fibrosis. (A) Representative immunohistochemical staining for ETAR, ETBR, collagen I and collagen III in infected livers. Black arrows indicate the positive cells. Scale bar, 100 μm. (B) Measurement of hepatic portal vein pressure in vivo by RM6240BD. (C) Statistical analysis of hepatic portal vein pressure (n = 5). (D) Analysis of portal vein diameter in vivo (n = 4). (E-F) Serum ALT and AST levels were measured (n = 5). (G-H) Liver and spleen indexes were determined (n = 4–6). (I-M) qPCR analysis of the expression levels of ET-1, ETAR, ETBR, Col1α1 and Col3α1 in liver samples (n = 3–6). (N-P) ETAR and ETBR proteins were determined by western blotting. Image density was quantified using Image J analysis and normalized to GAPDH (n = 5). Data are represented as mean ± SEM of three independent experiments. Significance was determined by the two-tailed Student’s t test. *P < 0.05, **P < 0.01, ***P < 0.001, ****P < 0.0001, compared with 0W samples. Abbreviation: ALT: alanine aminotransferase; AST: aspartate aminotransferase.
(TIF)

S5 Fig Reduced egg production of parasites in the hosts post praziquantel treatment. Mice were infected percutaneously with 16 *S*. *japonicum* cercariae or remained uninfected. At 6 weeks post-infection, the infected mice were treated with praziquantel to kill the parasites and then were necropsied at 12 weeks post-infection. (A) Macrograph of livers and spleens from uninfected mice, *S*. *japonicum* infected mice and infected mice treated with praziquantel. Scale bar, 1 cm. (B-D) The parasite living in the host and egg burden in the liver were counted (n = 5–6). Data are represented as mean ± SEM of three independent experiments. Significance was determined by the two-tailed Student’s t test. ****P < 0.0001, compared with Infected samples. Abbreviation: Sj: *Schistosoma japonicum*; PZQ: praziquantel.
(TIF)

S6 Fig Endothelin receptor antagonists moderated liver fibrosis in murine schistosomiasis and the alteration of SEA-specific IgG and egg burden. (A) Representative photographs of H&E stained sections of livers. Yellow dashed lines depicted the areas of liver fibrosis. Scale bar, 200 μm. (B) Quantification of the area of liver fibrosis (n = 7). (C) The serum level of SEA-specific IgG was assayed by ELISA (n = 7). (D) The egg burden in the liver was counted. Data are represented as mean ± SEM of three independent experiments. Multiple comparisons were performed by one-way ANOVA with Tukey’s correction for comparison between two groups.
(TIF)

S7 Fig Endothelin receptor antagonists partially altered the Th1/Th2 balance during infection. **(A-F)** The serum levels of IFN-γ, TNF, IL-2, IL-4, IL-5 and IL-13 were assayed by CBA (n = 4–7). Data are represented as mean ± SEM of three independent experiments. Multiple comparisons were performed by one-way ANOVA with Tukey’s correction for comparison between two groups.
(TIF)

S8 Fig ETRs were scantily located in the endothelial cells of the splenic sinus. Representative immunofluorescence staining of ETAR, ETBR and CD31^+^ endothelial cells in mice spleen tissues. Scale bar, 100 μm.
(TIF)

S9 Fig Purity of isolated B cells and CD4^+^CD25^-^ T cells. (A) Representative results for the B cell purification. (B) Representative results for the CD4^+^CD25^-^ T cell purification.
(TIF)

S10 Fig IL-10 production by CD19^+^ B cells during infection. Mouse CD19^+^ B cells were isolated from the spleen at different time points during schistosome infection. The B cells were cultured in the presence of SEA (20 μg/ml) for two days. Supernatants were stored for IL-10 analysis by ELISA (n = 6). Data are represented as mean ± SEM of three independent experiments. Significance was determined by the two-tailed Student’s t test. ***P< 0.001, ****P < 0.0001, compared with 0W samples.
(TIF)

S11 Fig Anti-CD20 treatment reduced levels of IL-10 in vitro. (A-C) Mice were injected with anti-CD20 to deplete B cells. After 7 days, isolated mouse splenic mononuclear cells were stimulated with SEA (20 μg/ml) for 3 days. (B) B cell (CD19^+^) frequency was determined in the spleen at 7 days post injection. (C) The splenic mononuclear cells were cultured in the presence of SEA (20 μg/ml) for 3 days. Supernatants were stored for IL-10 analysis by ELISA (n = 7). Data are represented as mean ± SEM of three independent experiments. Multiple comparisons were performed by one-way ANOVA with Tukey’s correction for comparison between two groups.
(TIF)

S12 Fig Representative photomicrographs showed tissue sections stained with control antibodies. Scale bar, 100 μm.
(TIF)
