# Supplementary figures and images for: Endothelin receptors promote schistosomiasis-induced hepatic fibrosis via splenic B cells
Source: PLoS Pathog. 2020 Oct 19;16(10):e1008947. doi: 10.1371/journal.ppat.1008947 (PMC7595619; doi:10.1371/journal.ppat.1008947)

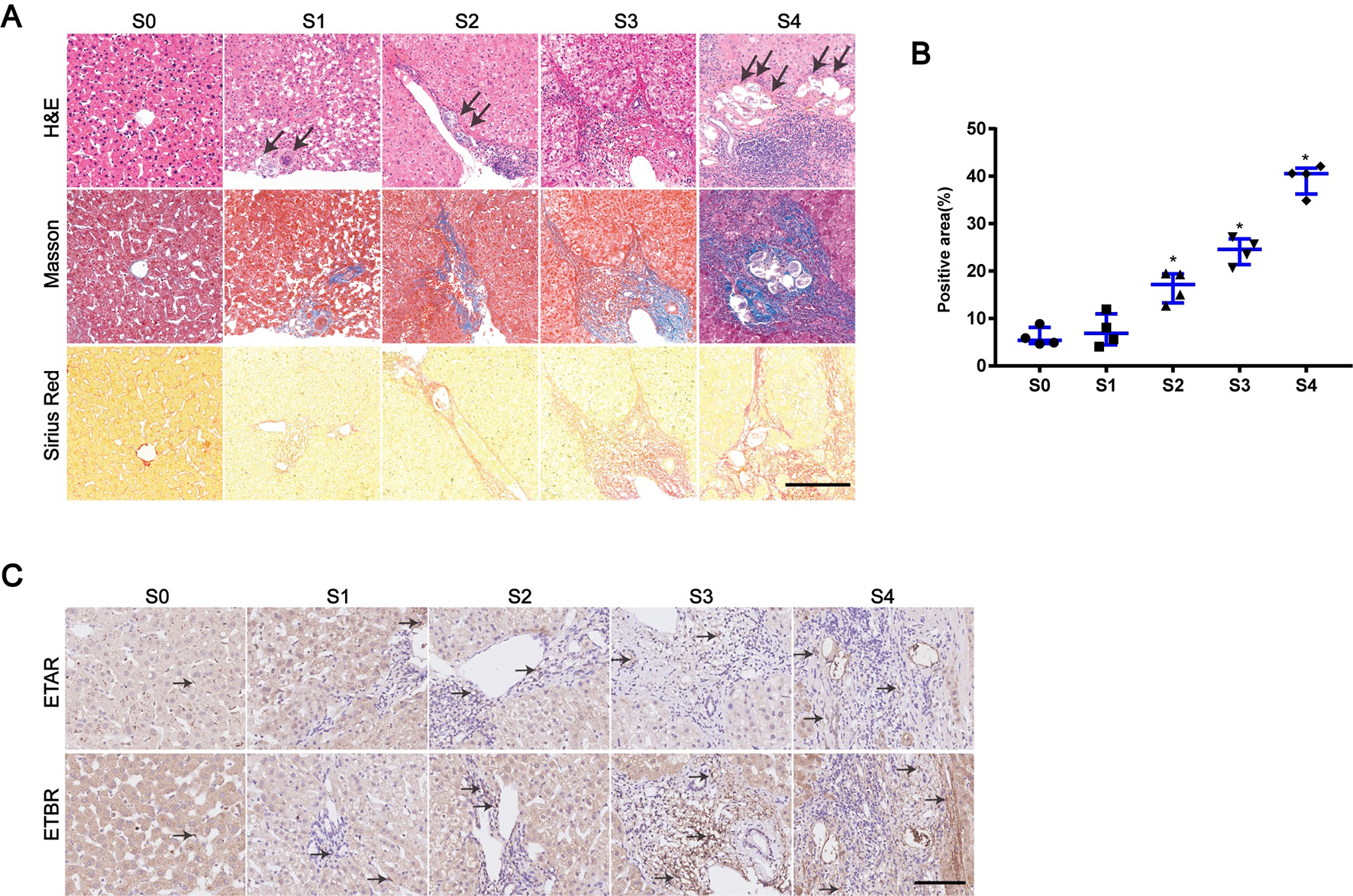

Supplement: S1 Fig — (A) Evaluation of the fibrotic stage in liver biopsy specimens by H&E and Masson’s trichrome staining. Paraffin-embedded sections of liver tissues from patients were stained with H&E, Masson’s trichrome and Sirius Red. Black arrows indicate worm eggs. Scale bar, 200 μm. (B) The positive staining areas for Sirius Red were measured using IPP software (n = 4). (C) Representative immunohistochemical staining of ETAR and ETBR. Black arrows indicate the ETRs positive cells. Scale bar, 100 μm. Data are represented as the median and interquartile range of two independent experiments. Significance was determined by the Mann-Whitney U-test. *P < 0.05, compared with S0 samples. (TIF) [file ppat.1008947.s001.tif]

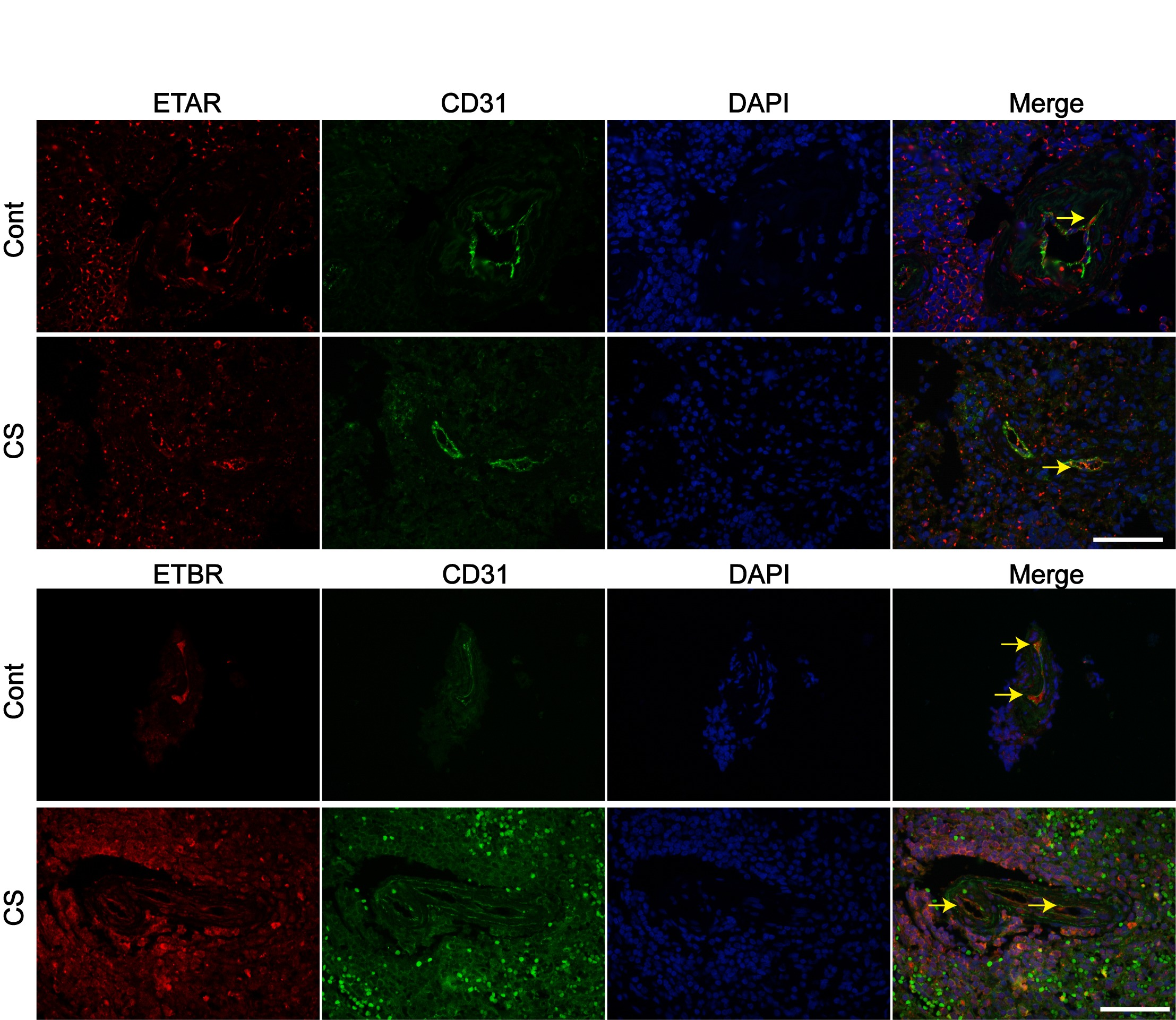

Supplement: S2 Fig — Representative immunofluorescence staining of ETAR, ETBR and CD31+ endothelial cells in human spleen tissues. Yellow arrows denote positive cells. Scale bar, 100 μm. (TIF) [file ppat.1008947.s002.tif]

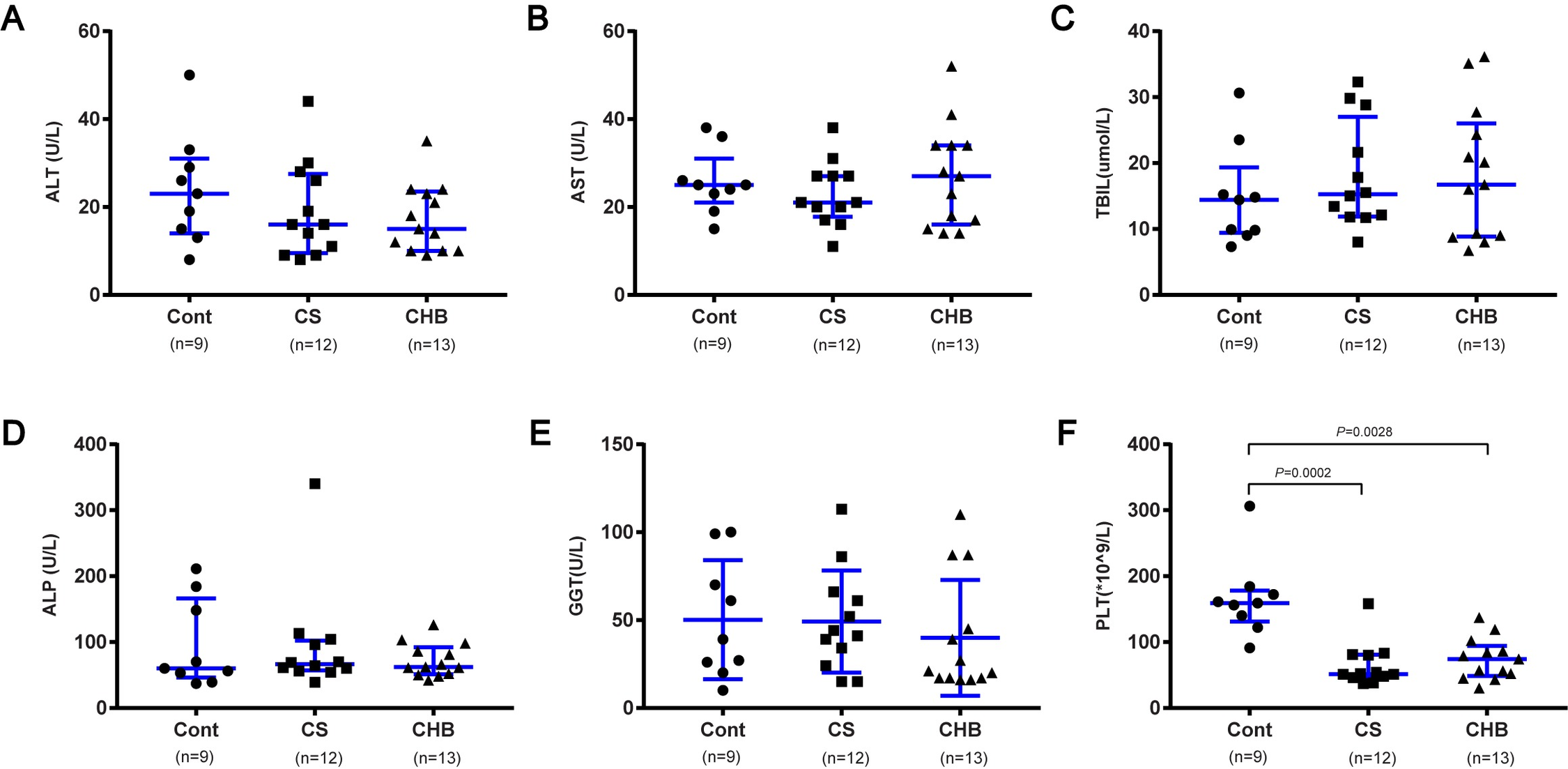

Supplement: S3 Fig — (A-F) ALT, AST, TBIL, ALP, GGT and PLT in patients who underwent splenectomy. Cont (n = 9), CS (n = 12), CHB (n = 13). Data are represented as the median and interquartile range. Significance was calculated using Kruskal–Wallis with Dunn’s posttest. Abbreviation: Cont, control; CS, chronic schistosomiasis; CHB, chronic hepatitis B; ALT: alanine aminotransferase; AST: aspartate aminotransferase; TBIL: total bilirubin; ALP: alkaline phosphatase; GGT: gamma-glutamyl transpeptidase; PLT: platelet. (TIF) [file ppat.1008947.s003.tif]

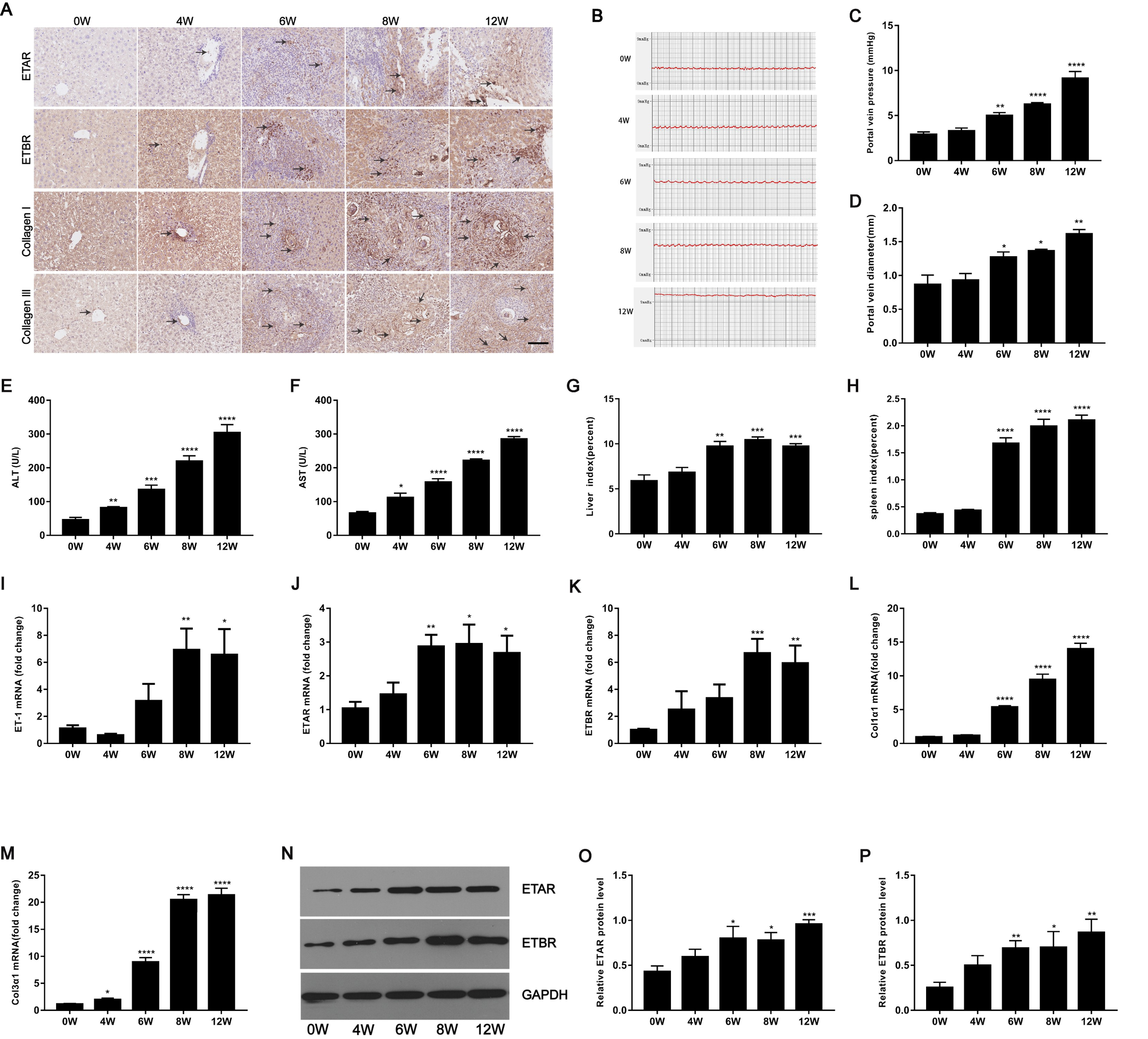

Supplement: S4 Fig — (A) Representative immunohistochemical staining for ETAR, ETBR, collagen I and collagen III in infected livers. Black arrows indicate the positive cells. Scale bar, 100 μm. (B) Measurement of hepatic portal vein pressure in vivo by RM6240BD. (C) Statistical analysis of hepatic portal vein pressure (n = 5). (D) Analysis of portal vein diameter in vivo (n = 4). (E-F) Serum ALT and AST levels were measured (n = 5). (G-H) Liver and spleen indexes were determined (n = 4–6). (I-M) qPCR analysis of the expression levels of ET-1, ETAR, ETBR, Col1α1 and Col3α1 in liver samples (n = 3–6). (N-P) ETAR and ETBR proteins were determined by western blotting. Image density was quantified using Image J analysis and normalized to GAPDH (n = 5). Data are represented as mean ± SEM of three independent experiments. Significance was determined by the two-tailed Student’s t test. *P < 0.05, **P < 0.01, ***P < 0.001, ****P < 0.0001, compared with 0W samples. Abbreviation: ALT: alanine aminotransferase; AST: aspartate aminotransferase. (TIF) [file ppat.1008947.s004.tif]

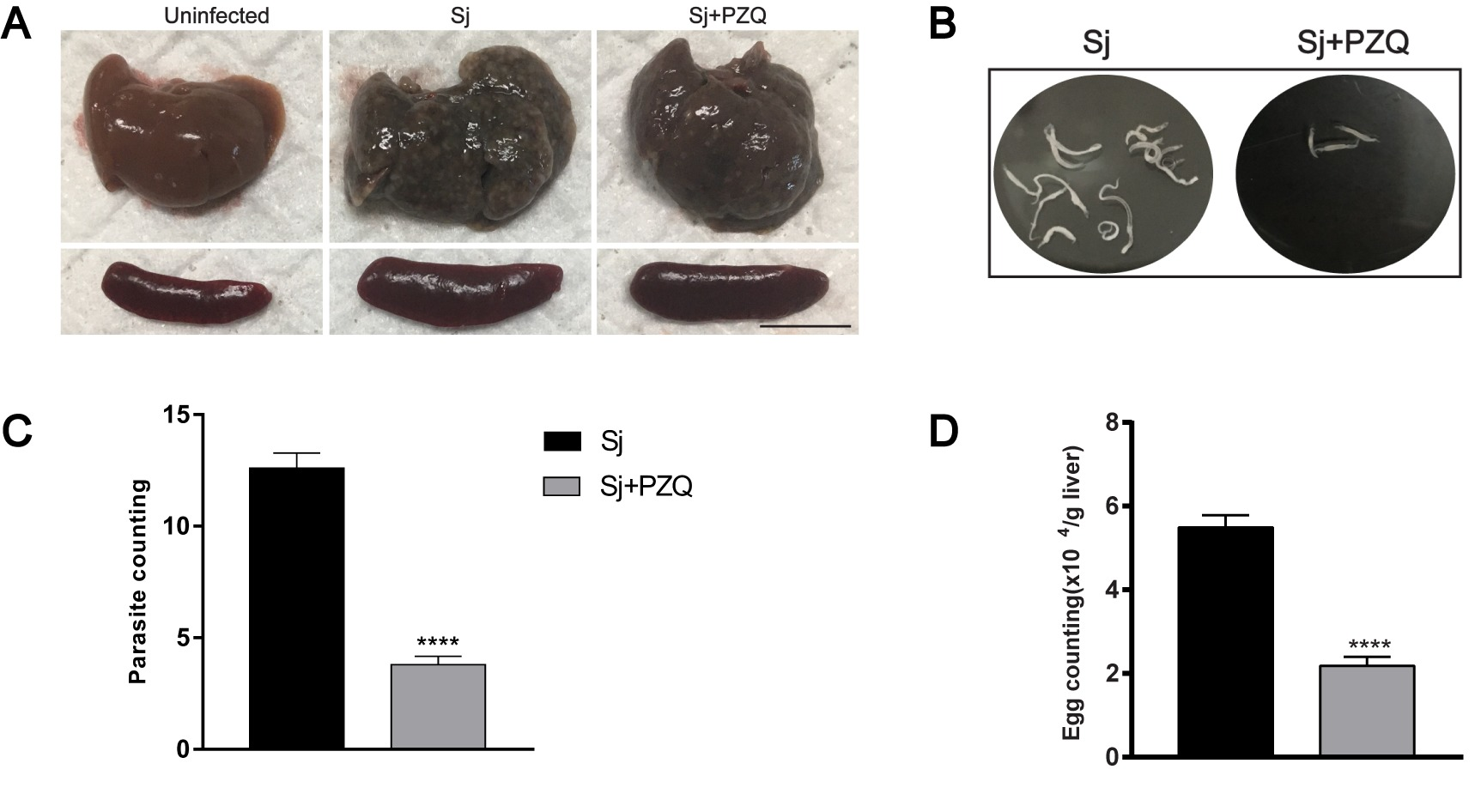

Supplement: S5 Fig — Mice were infected percutaneously with 16 S. japonicum cercariae or remained uninfected. At 6 weeks post-infection, the infected mice were treated with praziquantel to kill the parasites and then were necropsied at 12 weeks post-infection. (A) Macrograph of livers and spleens from uninfected mice, S. japonicum infected mice and infected mice treated with praziquantel. Scale bar, 1 cm. (B-D) The parasite living in the host and egg burden in the liver were counted (n = 5–6). Data are represented as mean ± SEM of three independent experiments. Significance was determined by the two-tailed Student’s t test. ****P < 0.0001, compared with Infected samples. Abbreviation: Sj: Schistosoma japonicum; PZQ: praziquantel. (TIF) [file ppat.1008947.s005.tif]

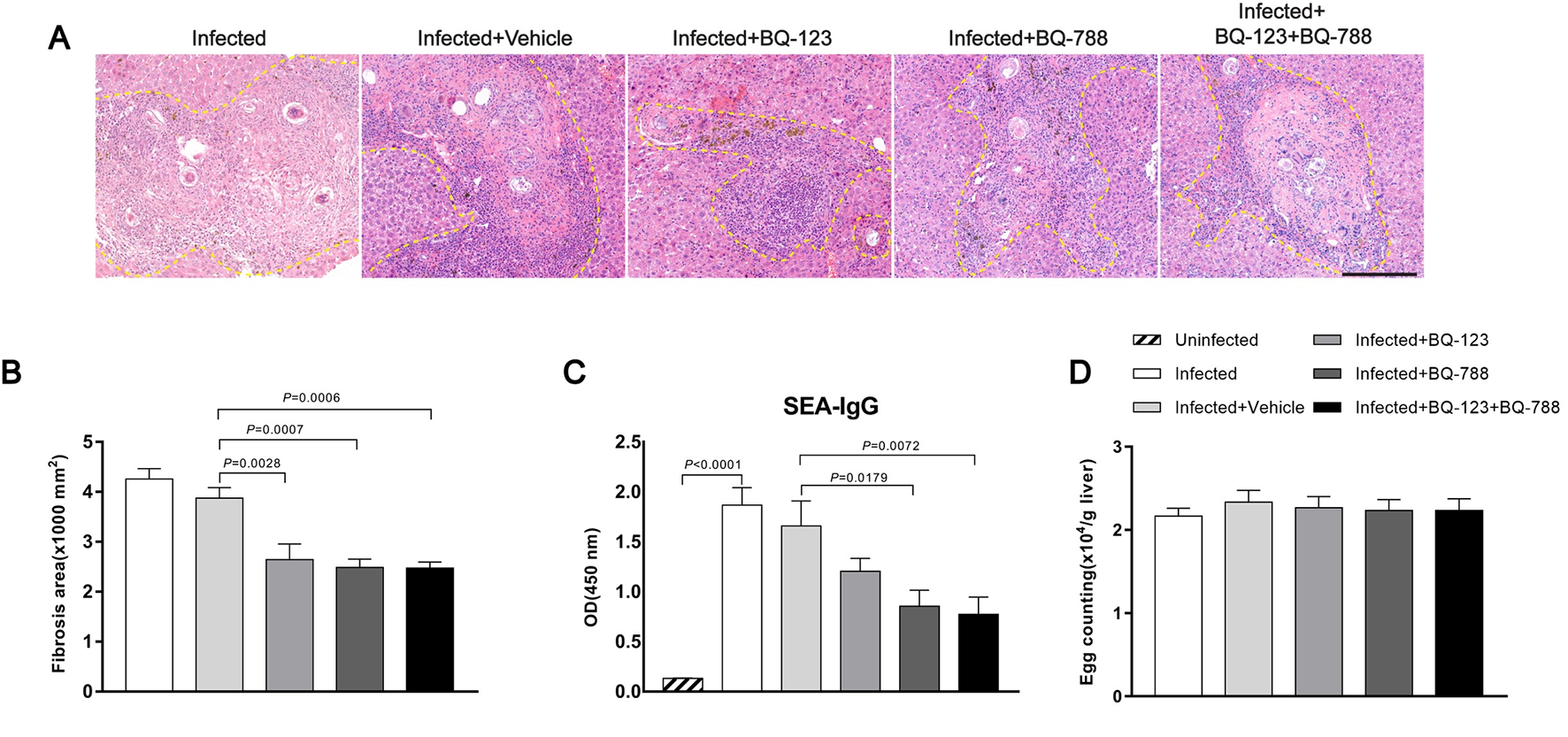

Supplement: S6 Fig — (A) Representative photographs of H&E stained sections of livers. Yellow dashed lines depicted the areas of liver fibrosis. Scale bar, 200 μm. (B) Quantification of the area of liver fibrosis (n = 7). (C) The serum level of SEA-specific IgG was assayed by ELISA (n = 7). (D) The egg burden in the liver was counted. Data are represented as mean ± SEM of three independent experiments. Multiple comparisons were performed by one-way ANOVA with Tukey’s correction for comparison between two groups. (TIF) [file ppat.1008947.s006.tif]

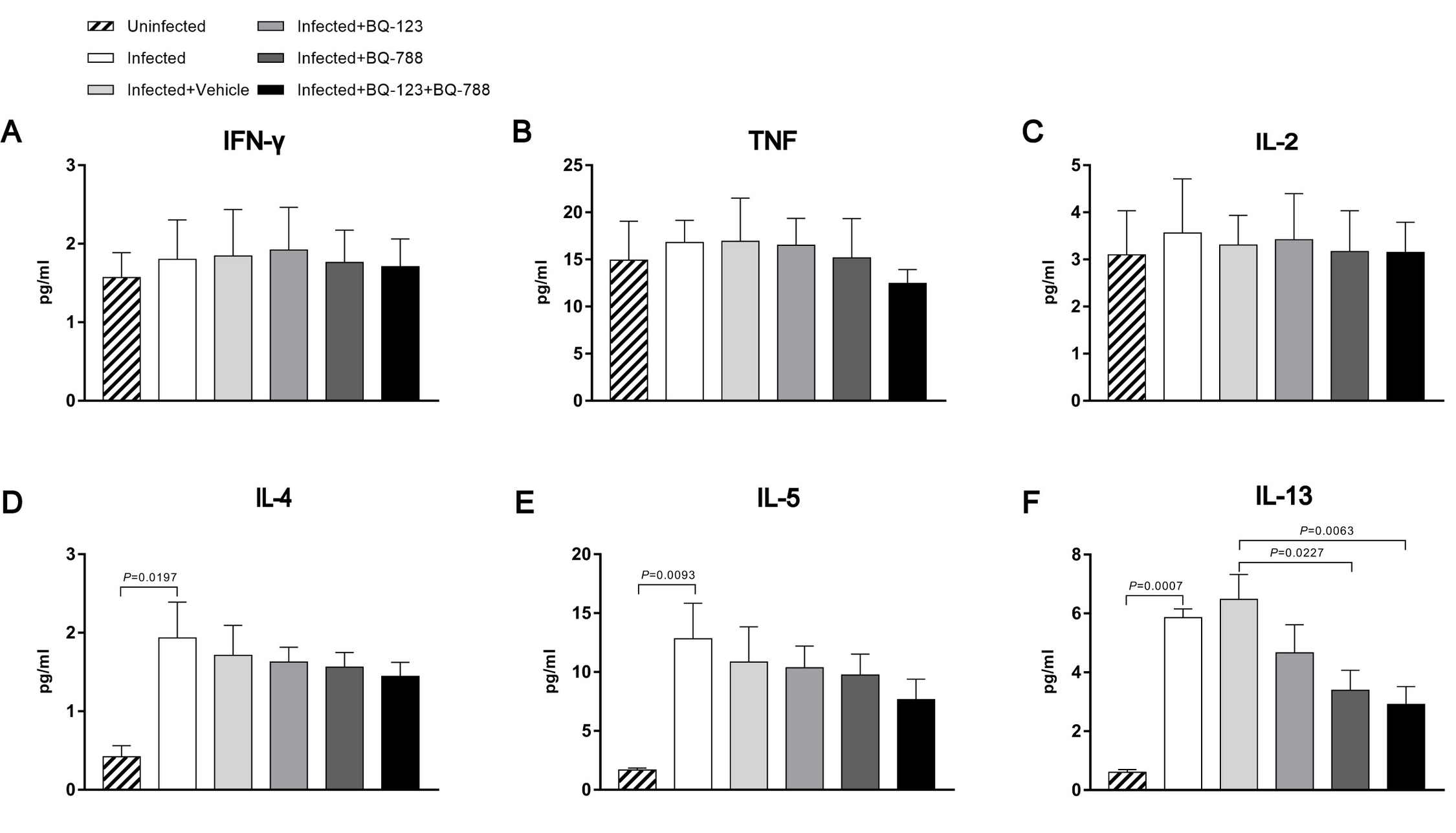

Supplement: S7 Fig — (A-F) The serum levels of IFN-γ, TNF, IL-2, IL-4, IL-5 and IL-13 were assayed by CBA (n = 4–7). Data are represented as mean ± SEM of three independent experiments. Multiple comparisons were performed by one-way ANOVA with Tukey’s correction for comparison between two groups. (TIF) [file ppat.1008947.s007.tif]

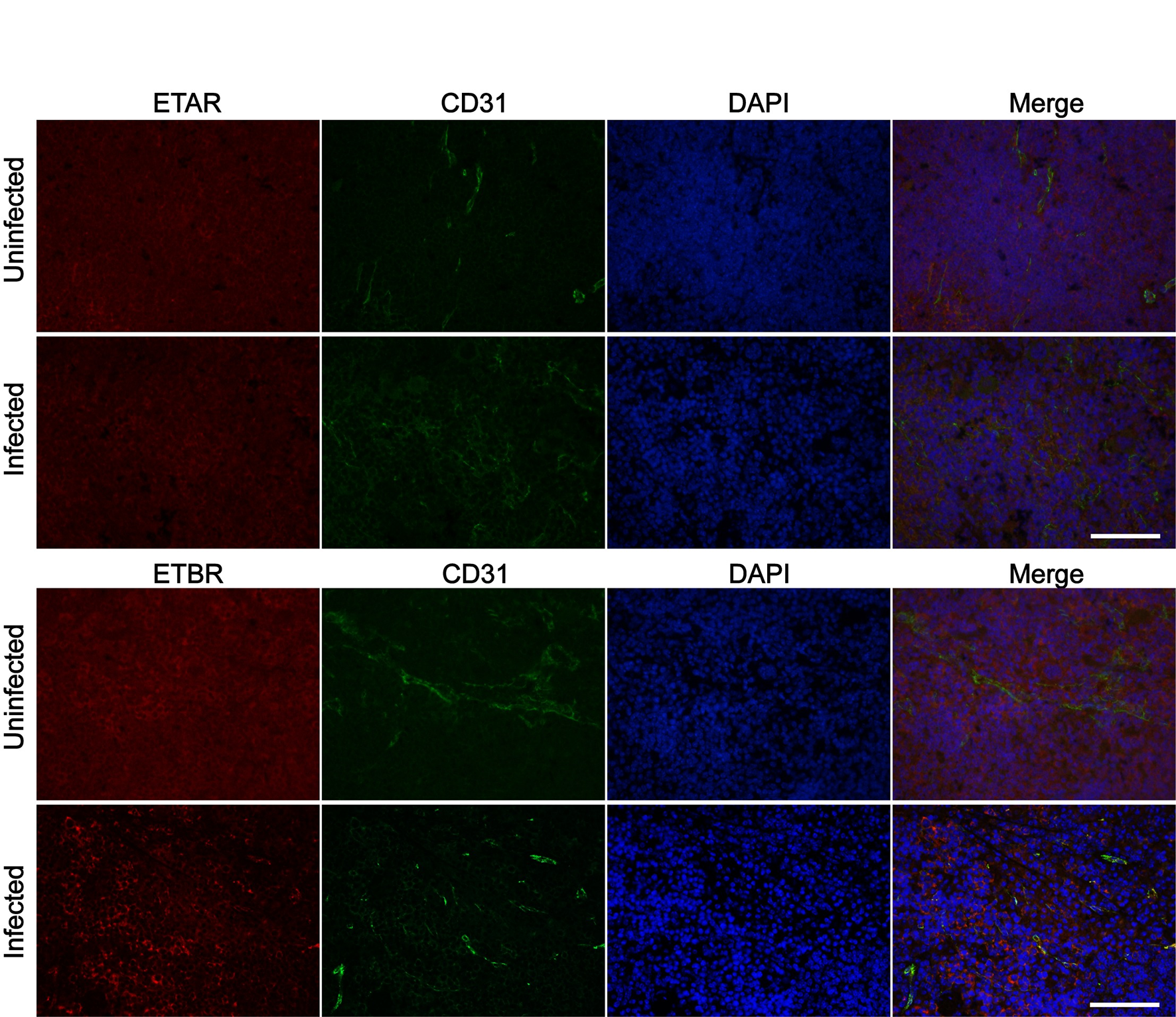

Supplement: S8 Fig — Representative immunofluorescence staining of ETAR, ETBR and CD31+ endothelial cells in mice spleen tissues. Scale bar, 100 μm. (TIF) [file ppat.1008947.s008.tif]

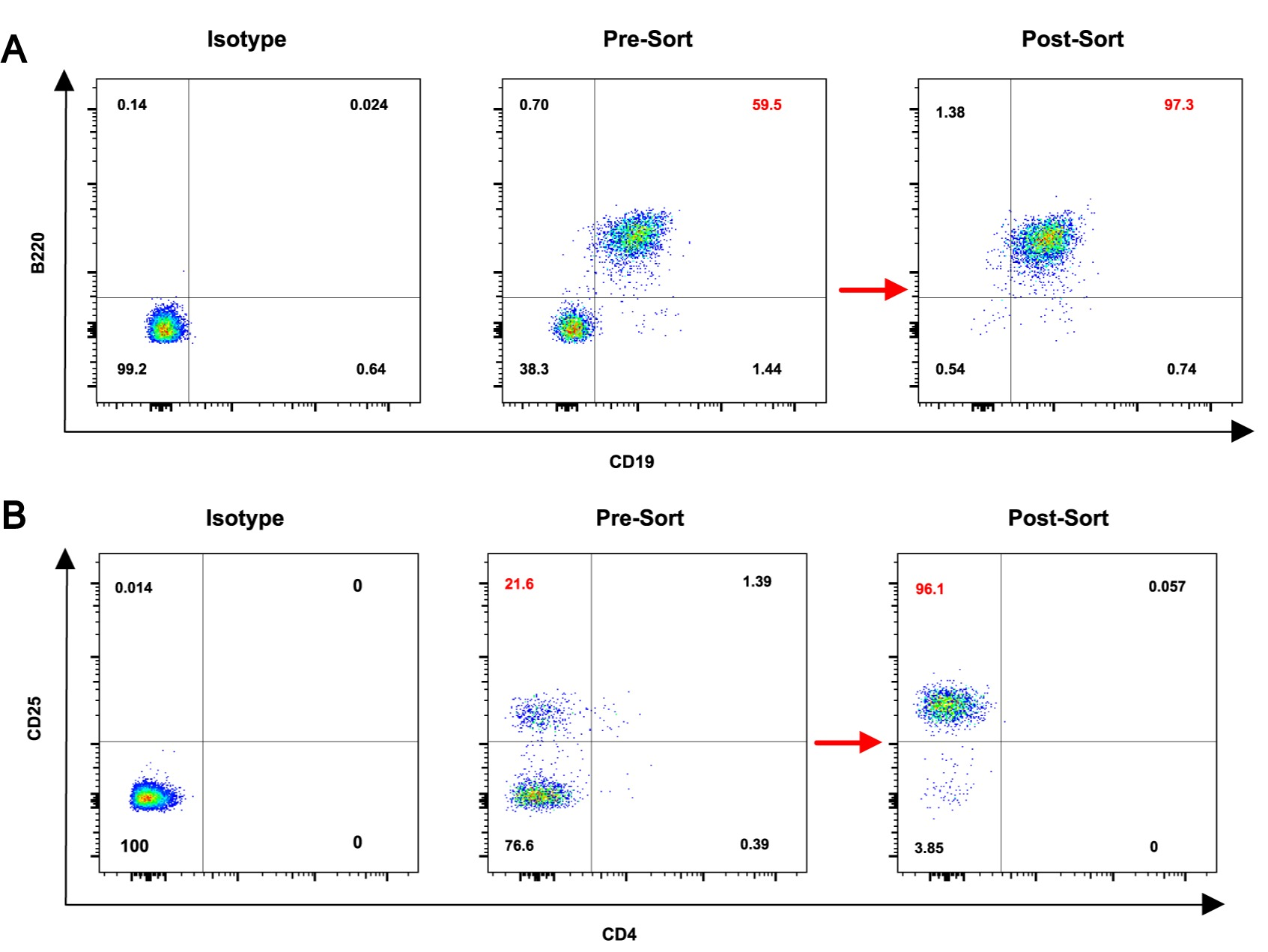

Supplement: S9 Fig — (A) Representative results for the B cell purification. (B) Representative results for the CD4+CD25- T cell purification. (TIF) [file ppat.1008947.s009.tif]

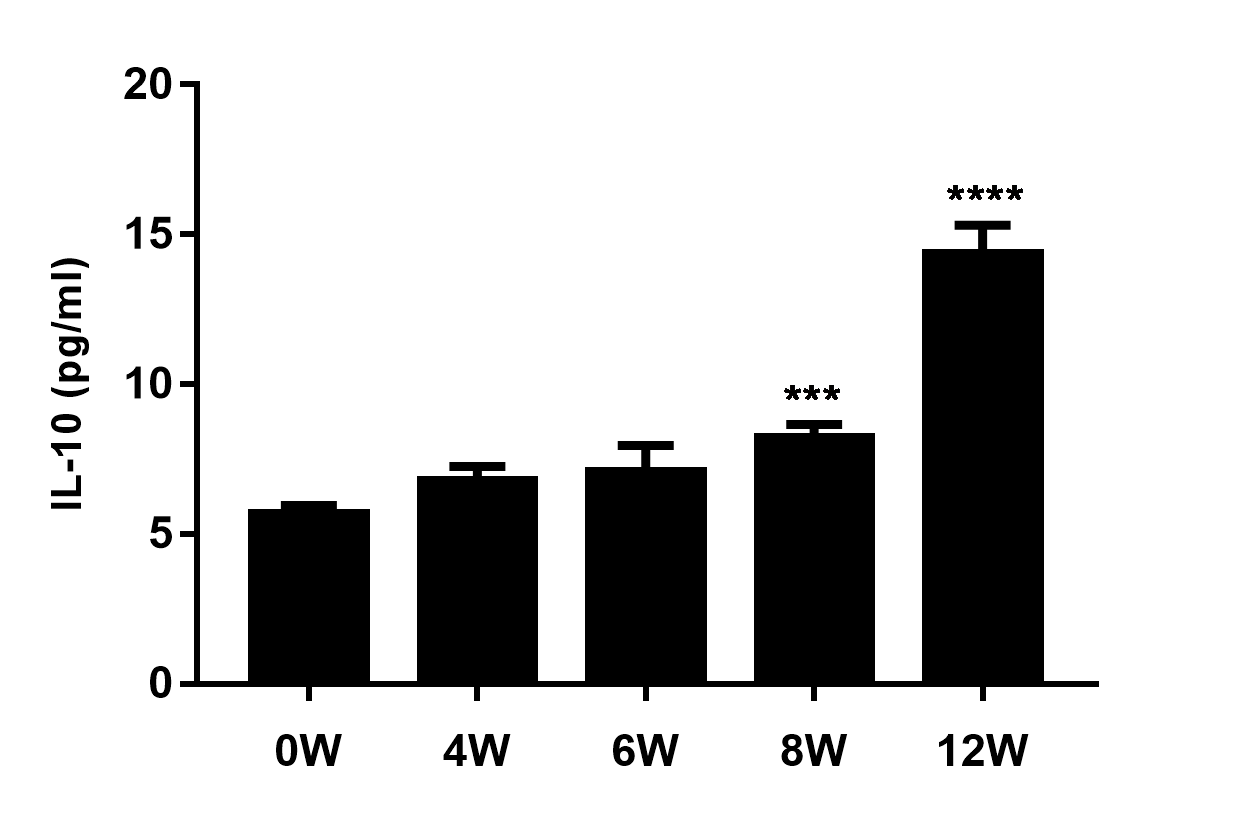

Supplement: S10 Fig — Mouse CD19+ B cells were isolated from the spleen at different time points during schistosome infection. The B cells were cultured in the presence of SEA (20 μg/ml) for two days. Supernatants were stored for IL-10 analysis by ELISA (n = 6). Data are represented as mean ± SEM of three independent experiments. Significance was determined by the two-tailed Student’s t test. ***P< 0.001, ****P < 0.0001, compared with 0W samples. (TIF) [file ppat.1008947.s010.tif]

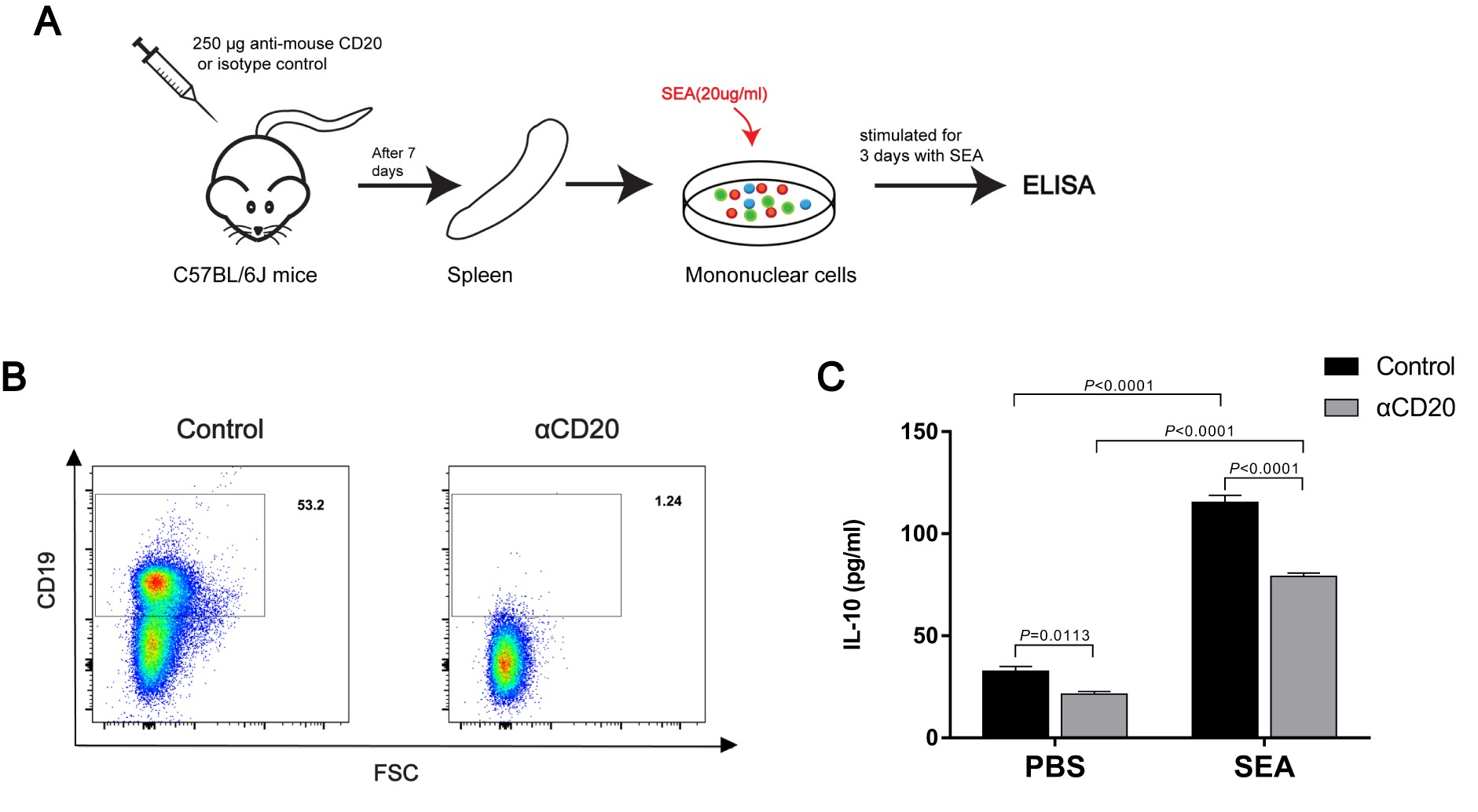

Supplement: S11 Fig — (A-C) Mice were injected with anti-CD20 to deplete B cells. After 7 days, isolated mouse splenic mononuclear cells were stimulated with SEA (20 μg/ml) for 3 days. (B) B cell (CD19+) frequency was determined in the spleen at 7 days post injection. (C) The splenic mononuclear cells were cultured in the presence of SEA (20 μg/ml) for 3 days. Supernatants were stored for IL-10 analysis by ELISA (n = 7). Data are represented as mean ± SEM of three independent experiments. Multiple comparisons were performed by one-way ANOVA with Tukey’s correction for comparison between two groups. (TIF) [file ppat.1008947.s011.tif]

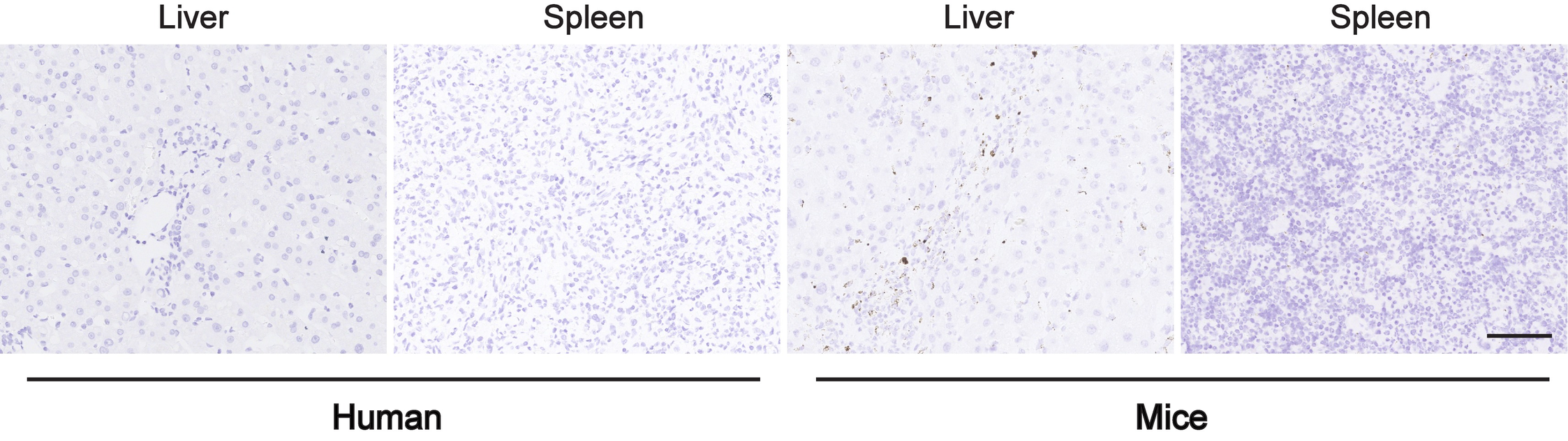

Supplement: S12 Fig — Scale bar, 100 μm. (TIF) [file ppat.1008947.s012.tif]
